# The Transcription Factor Nfatc2 Regulates β-Cell Proliferation and Genes Associated with Type 2 Diabetes in Mouse and Human Islets

**DOI:** 10.1371/journal.pgen.1006466

**Published:** 2016-12-09

**Authors:** Mark P. Keller, Pradyut K. Paul, Mary E. Rabaglia, Donnie S. Stapleton, Kathryn L. Schueler, Aimee Teo Broman, Shuyun Isabella Ye, Ning Leng, Christopher J. Brandon, Elias Chaibub Neto, Christopher L. Plaisier, Shane P. Simonett, Melkam A. Kebede, Gloria M. Sheynkman, Mark A. Klein, Nitin S. Baliga, Lloyd M. Smith, Karl W. Broman, Brian S. Yandell, Christina Kendziorski, Alan D. Attie

**Affiliations:** 1 Department of Biochemistry, University of Wisconsin, Madison, Wisconsin, United States of America; 2 Department of Biostatistics & Medical Informatics, University of Wisconsin, Madison, Wisconsin, United States of America; 3 Department of Statistics, University of Wisconsin, Madison, Wisconsin, United States of America; 4 Sage Bionetworks, Seattle, Washington; 5 Institute for Systems Biology, Seattle, Washington; 6 Department of Chemistry, University of Wisconsin, Madison, Wisconsin, United States of America; UCLA School of Medicine, UNITED STATES

## Abstract

Human genome-wide association studies (GWAS) have shown that genetic variation at >130 gene loci is associated with type 2 diabetes (T2D). We asked if the expression of the candidate T2D-associated genes within these loci is regulated by a common locus in pancreatic islets. Using an obese F2 mouse intercross segregating for T2D, we show that the expression of ~40% of the T2D-associated genes is linked to a broad region on mouse chromosome (Chr) 2. As all but 9 of these genes are not physically located on Chr 2, linkage to Chr 2 suggests a genomic factor(s) located on Chr 2 regulates their expression in *trans*. The transcription factor *Nfatc2* is physically located on Chr 2 and its expression demonstrates *cis* linkage; *i*.*e*., its expression maps to itself. When conditioned on the expression of *Nfatc2*, linkage for the T2D-associated genes was greatly diminished, supporting *Nfatc2* as a driver of their expression. Plasma insulin also showed linkage to the same broad region on Chr 2. Overexpression of a constitutively active (ca) form of *Nfatc2* induced β-cell proliferation in mouse and human islets, and transcriptionally regulated more than half of the T2D-associated genes. Overexpression of either ca-Nfatc2 or ca-Nfatc1 in mouse islets enhanced insulin secretion, whereas only ca-Nfatc2 was able to promote β-cell proliferation, suggesting distinct molecular pathways mediating insulin secretion *vs*. β-cell proliferation are regulated by NFAT. Our results suggest that many of the T2D-associated genes are downstream transcriptional targets of NFAT, and may act coordinately in a pathway through which NFAT regulates β-cell proliferation in both mouse and human islets.

## Author Summary

Genome-wide association studies (GWAS) and linkage studies provide a powerful way to establish a causal connection between a gene locus and a physiological or pathophysiological phenotype. We wondered if candidate genes associated with type 2 diabetes in human populations, in addition to being causal for the disease, could also be intermediate traits in a pathway leading to disease. In addition, we wished to know if there were any regulatory loci that could coordinately drive the expression of these genes in pancreatic islets and thus complete a pathway; i.e. Driver → GWAS candidate expression → type 2 diabetes. Using data from a mouse intercross between a diabetes-susceptible and a diabetes-resistant mouse strain, we found that the expression of ~40% of >130 candidate GWAS genes genetically mapped to a hot spot on mouse chromosome 2. Using a variety of statistical methods, we identified the transcription factor *Nfatc2* as the candidate driver. Follow-up experiments showed that overexpression of *Nfatc2* does indeed affect the expression of the GWAS genes and regulates β-cell proliferation and insulin secretion. The work shows that in addition to being causal, GWAS candidate genes can be intermediate traits in a pathway leading to disease. Model organisms can be used to explore these novel causal pathways.

## Introduction

Human genome-wide association studies (GWAS) have identified genetic variation at >130 loci that is associated with various traits linked to the development of T2D. The vast majority of the T2D-associated SNPs occur in intergenic or intronic regions, implying that they are involved in gene regulation. Specific candidate genes have been suggested to mediate the association between the T2D-associated SNPs and T2D risk. Recent studies have shown that many of the T2D-associated candidate genes primarily affect the health and function of pancreatic islet cells [1]. Thus, understanding the relationship between their functional role in pancreatic islets, and proximity to T2D-associated loci is critically important.

Genetic association is defined as a causal relationship between genetic variation at a locus and a disease phenotype. However, despite the large number of loci that are associated with T2D, collectively they account for only ~10% of the genetic variability of the disease [2]. This “hidden heritability” has been attributed to the inability to detect rare alleles and/or epistasis [3]. It is also possible that dysregulation of pathways leading to T2D occur in many different ways and thus do not reflect genetic variation at a single locus.

We and others have explored the high heritability of mRNA abundance [4–7]. In these studies, we have identified expression quantitative trait loci (eQTLs), which control the expression level or abundance of one or more mRNAs as quantitative traits. These traits show high heritability and provide a means to identify sets of co-regulated genes that are often associated with common physiological pathways. When the expression of a group of mRNAs map to a common locus, we can hypothesize that the associated genes are co-regulated at that locus.

We previously identified eQTLs in an obese F2 mouse population derived from diabetes-susceptible and diabetes-resistant founder strains [8]. We performed genome-wide expression profiling from pancreatic islets and determined the genetic architecture of all islet mRNA transcripts showing heritability in this F2 population. Approximately 10% of the transcripts that showed genetic regulation in mouse islets, demonstrated linkage to the region in the genome where the gene encoding the transcript physically resides; *i*.*e*. *cis* or proximal eQTLs. The remaining ~90% mapped to a region where the encoding gene does not reside *(i*.*e*. *trans* or distal eQTLs). Often, *trans*-eQTLs co-mapped to a common locus, giving rise to *trans*-eQTL hot spots that potentially link to a common regulator. We recently used this approach to identify a bile acid transporter in pancreatic islets as a strong candidate for an islet eQTL hot spot on the distal end of Chr 6 [8].

Here we asked if the expression of T2D-associated candidate genes from human GWAS are subject to coordinate regulation in pancreatic islets. Our model system was an F2 intercross between a diabetes-resistant (B6) and a diabetes-susceptible (BTBR) mouse strain [9]. Approximately 500 F2 mice were made genetically obese in response to the leptin mutation (*Lep*^*ob/ob*^), and all mice were sacrificed at 10 weeks of age in order to collect their pancreatic islets for transcriptomic profiling. An interactive database of our gene expression and diabetes-related clinical phenotypes is provided at http://diabetes.wisc.edu/.

We identified *Nfatc2* as a strong candidate for the transcriptional regulation of the T2D-associated genes, and responsible for an underlying islet eQTL hot spot on Chr 2. Many of the T2D-associated candidate genes whose expression mapped in *trans* to a broad region on Chr 2, were regulated by *Nfatc2* in both mouse and human islets. In addition to transcriptionally regulating the expression of a large number of the GWAS gene candidates, the overexpression of *Nfatc2* induced β-cell proliferation. Our results suggest that *Nfatc2* is a key regulator of β-cell proliferation and function that may in part reflect the coordinate regulation within islets of candidate genes associated with T2D in human studies.

## Results

### Identification of gene candidates linked to T2D from human GWAS

In order to ask if T2D-associated gene candidates from human studies showed coordinate regulation in mouse islets, we first generated a list of gene candidates, and identified their mouse homologues**.** A catalog of published human GWAS loci was downloaded from the National Human Genome Research Institute (http://ebi.ac.uk/gwas). Approximately 300 separate entries (corresponding to 136 distinct genomic loci) were associated with the Disease/Trait, “Type 2 diabetes”, resulting in a list of 150 reported genes (S1 Table). We identified 141 mouse homologues to these human genes, 129 of which were represented on the gene array used for the islet eQTL analysis in our F2 mouse study. We emphasize that these are reported gene candidates that are linked to the genomic loci associated to human T2D risk. Importantly, a disease-associated SNP may not necessarily influence the nearest gene, as is the case for obesity-associated SNPs within the *FTO* gene, which appear to influence the expression of a more distal gene, *IRX3* [10, 11]. However, for our eQTL analysis, we included all genes reported to be associated with T2D.

### Chr 2 is a hotspot for regulation of the T2D GWAS candidates in mouse

**Fig 1:**
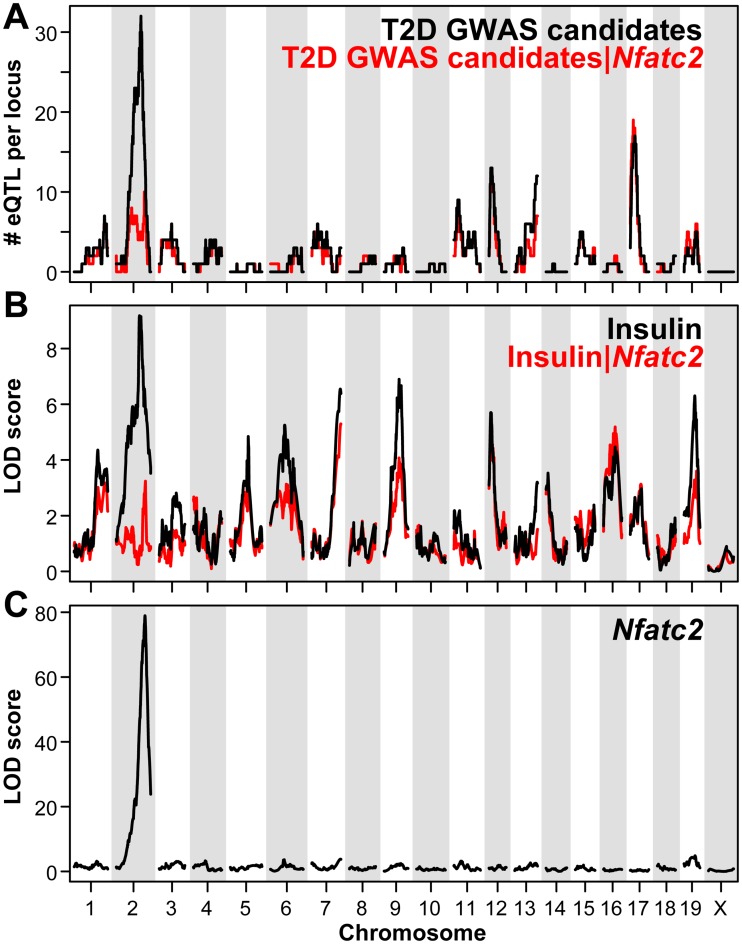
Genetic architecture of the islet expression of T2D GWAS gene candidates, plasma insulin, and *Nfatc2* in mice segregating for T2D. Panels illustrate the genome-wide LOD profiles for the islet expression of T2D GWAS gene candidates (**A**), fasting plasma insulin (**B**), or the islet expression of *Nfatc2* (**C**) in ~500 obese F2:B6-BTBR mice. T2D GWAS eQTLs are shown as the number of traits demonstrating linkage (LOD ≥ 5) to the same genomic locus (1.5 LOD support interval). Red traces in **A** and **B** illustrate QTLs for T2D GWAS gene candidates or plasma insulin, respectively, after conditioning on the *Nfatc2* *cis*-eQTL.

Approximately 60% of the transcripts encoded by mouse homologues of the T2D human GWAS candidates showed a significant eQTL (LOD ≥ 5) at one or more loci across the genome (Fig 1A). The vast majority of these eQTLs were distal, or *trans*-eQTLs, implying a regulatory factor controlling the expression of each transcript (S2 Table). Of the 205 eQTLs identified genome-wide, ~88% were *trans*-eQTLs with an average LOD score of ~9 (genome-wide *P* = 0.05). There were 25 eQTLs that were proximal, or *cis* to the encoding gene; the average LOD score for these *cis*-eQTL was ~40. Four loci were identified where >10 GWAS-associated eQTLs co-mapped to the same locus; chromosomes 2, 12, 13 and 17. A broad locus on Chr 2 showed the greatest number of co-mapping eQTLs, corresponding to >50 GWAS candidate genes.

The gene that has the strongest association with T2D in humans is *TCF7L2* [12]. In the mouse, the gene for *Tcf7l2* is located on Chr 19 (Chr 10 in human), yet its expression maps in *trans* to Chr 2 at ~148 Mbp with a LOD score of ~9. Co-mapping with *Tcf7l2*, were ~50 other GWAS candidate genes, the majority of them mapping in *trans* to a broad region on Chr 2 (S1 Fig). The genotype dependence of the GWAS eQTLs showed that approximately half of them decreased expression with BTBR alleles; the remaining half showed increased expression with BTBR alleles (S2 Fig). In addition to the *trans*-eQTLs for the GWAS candidate genes, the strongest linkage to fasting plasma insulin in our F2 intercross occurred at a locus on Chr 2, ~148 Mb with a LOD of ~10 (Fig 1B).

### Nfatc2 is the top candidate regulator of the T2D GWAS candidates

One possible explanation for a group of genes demonstrating co-mapping *trans*-eQTLs is that the expression of a transcription factor is perturbed by a *cis*-acting variant, which in turn leads to a change in the expression of its target genes. The gene for the transcription factor *Nfatc2* is located at ~168 Mbp on Chr 2 and demonstrates a strong *cis*-eQTL with a LOD of ~80 (Fig 1C). To test whether variation in the GWAS candidate eQTLs can be explained by variation in *Nfatc2* expression, we considered a conditional QTL mapping analysis. In a conditional QTL analysis, a LOD profile is considered after regressing out the effect of another factor (e.g. another QTL or covariate) to assess the effect of the LOD profile once the effect of the factor has been removed. Specifically, for each GWAS eQTL, we first evaluated the LOD profile after regressing out the effect of *Nfatc2* expression. Upon doing so, the LOD score for 49 of the 54 GWAS-associated eQTLs was reduced on average by ~6 units (Fig 1A; see also S2 Table). We also evaluated the LOD profile of insulin after regressing out the effect of *Nfatc2* expression and again observed a significant decrease in the LOD profile for insulin (Fig 1B; see also S1B Fig). The dependence of the *trans*-eQTLs for the GWAS genes and the insulin QTL on *Nfatc2* is consistent with a pathway by which *Nfatc2* regulates the expression of the GWAS genes, which in turn influences plasma insulin in our obese B6:BTBR F2 mice.

To determine the specificity of Nfatc2’s effect on the GWAS eQTLs, we evaluated the potential influence of other transcription factors that demonstrated *cis* linkage on Chr 2. There are 36 genes with *cis*-eQTLs on Chr 2 that are known transcription factors, or annotated as playing a role in gene regulation (*e*.*g*., transcription or DNA binding). Conditional scans for the GWAS eQTLs were separately performed on each of these Chr 2 *cis*-eQTLs, and an overall influence score was computed. *Nfatc2* was the top-ranked transcription factor for having the largest impact on the co-mapping GWAS eQTLs (S3 Table). Our conditional analysis therefore supports *Nfatc2* as the most likely regulatory gene candidate mediating the *trans*-QTLs for the GWAS genes, as well as the QTL for plasma insulin.

The expression of *Nfatc2* is strongly linked to a single nucleotide polymorphism (SNP; rs3024096, ~167 Mb) between B6 and BTBR. This SNP results in reduced expression of *Nfatc2*, associated with the BTBR allele, yielding a ~2-fold difference between homozygous B6 *vs*. homozygous BTBR at the *Nfatc2* gene locus (S3 Fig). Further, sequencing of the BTBR genome identified two SNPs within the *Nfatc2* gene that yield a change in protein sequence relative to B6. A highly conserved proline (Pro^251^) is converted to a leucine (rs259322485), and a leucine (Leu^267^) is converted to a proline (rs27289000). These two residues flank the nuclear localization sequence, and thus may affect nuclear import of Nfatc2 following de-phosphorylation by calcineurin.

### NFATC2 is associated with human diabetes

Using LocusZoom [13], a visualization tool of publically-available GWAS data, we asked if SNPs near human *NFATC2* and *NFATC1* genes are associated with diabetes-related phenotypes (S4 Fig). Within a small interval of both genes, SNPs were identified that showed nominal association with fasting insulin levels in >50,000 non-diabetic individuals [14]. The nominal P-value for the SNPs associated with fasting insulin are ~10^−4^–10^−5^; these would not reach statistical significance once corrected for a genome-wide query, which usually require P-values of less than 5 x 10^−8^. However, by performing queries for a small number of SNPs, the penalty for multiple tests is greatly diminished, suggesting the fasting insulin-associated SNPs at the NFAT loci are significant. Our results showing that *trans*-eQTL linkages of the GWAS candidate genes to the *Nfatc2* locus in mouse is consistent with *NFATC2* association with diabetes traits in humans.

Previously, we performed RNA-sequencing of mouse islets with sufficient depth to quantify isoform-specific expression of all genes [15]. Islets from B6 mice express 3 isoforms of *Nfatc2*; variants 1, 2 and 4 in relative proportions of ~41%, ~5% and ~54% respectively (S4 Table). Six isoforms of *Nfatc1* are expressed (variants 1–6), with variants 1, 5 and 6 comprising >99% of the expressed isoforms. The most abundantly expressed *Nfatc1* variant (variant 1) is a smaller protein (703 amino acids) than the two abundantly expressed isoforms of *Nfatc2* (923 and 927 for variants 4 and 1 respectively). Among the four NFATc genes, *Nfatc3* showed the highest expression, whereas *Nfatc4* showed the lowest expression.

### Constitutively active NFAT induces β-cell proliferation

We examined the effects of the ca-NFATs on β-cell proliferation, insulin secretion, and the regulation of gene expression, including the T2D GWAS genes, in isolated mouse and human islets. We used the well-characterized constitutively active (ca) form of *Nfatc2*, where 12 serine residues are mutated to alanine in the N-terminal regulatory domain of the protein. In addition, we evaluated the effects of ca-Nfatc1, which had 17 serine residues mutated to alanine [16, 17]. We employed adenoviruses to overexpress the constitutively active mutants of mouse *Nfatc1* and *Nfatc2*. To mimic the relative abundance of the endogenously expressed NFAT isoforms, the viruses were generated from variant 1 for each gene; 703 amino acids for *Nfatc1* and 927 amino acids for *Nfatc2*. The protein sequences for the corresponding isoforms of mouse and human Nfatc1 and Nfatc2 are ~86% and ~90% identical respectively; all of the Ser residues that were mutated to Ala residues in each of the ca-NFAT mutants are identical between mouse and human (S5 Fig).

The adenoviruses increased the expression (S6A Fig) and protein levels (S6B Fig) of ca-Nfatc1 and ca-Nfatc2 in mouse islets. The overexpression of either ca-mutant did not induce the expression of the other; *i*.*e*., ca-Nfatc1 did not induce endogenous *Nfatc2* and ca-Nfatc2 did not induce endogenous *Nfatc1*. The expression of *Nfatc4*, the NFATc gene with the lowest overall endogenous expression, was induced ~3.5 (*P* < 10^−4^) and 4.8-fold (*P* < 0.02) in response to ca-Nfatc1 and ca-Nfatc2 respectively, although it remained the lowest expressed gene, despite the induction evoked by the ca-NFATs.

**Fig 2:**
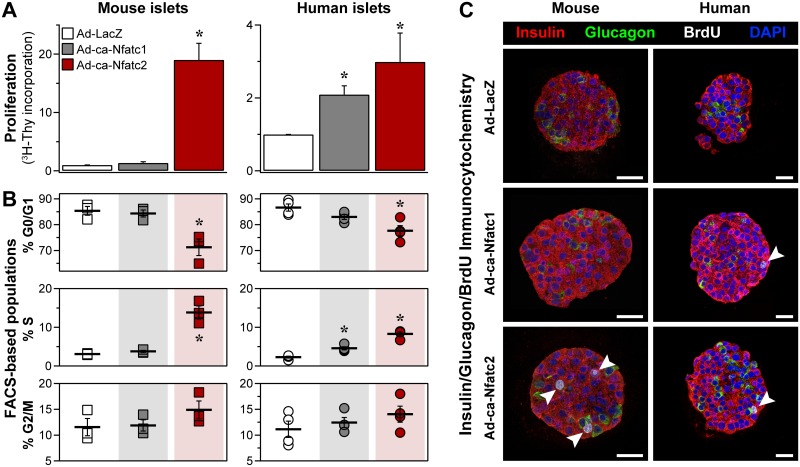
NFAT triggers β-cell proliferation in mouse and human islets. Adenoviruses (Ad) were used to overexpress constitutively active (ca) *Nfatc1* or *Nfatc2* in human and mouse islets; Ad-LacZ was used as the negative control. Cellular proliferation was monitored by incorporation of [^3^H]-thymidine into islet DNA (**A**), FACS-based analysis of cell cycle phases (**B**), and incorporation of BrdU (white arrow heads) into islet cells that were co-stained for insulin or glucagon to identify β-cells and α-cells respectively (**C**). Thymidine incorporation measurements were conducted on 5 and 3 separate mouse and human islet preparations, respectively. Immunofluorescent images are representative of >30 islets (BrdU) per adenoviral treatment collected from 5 mice, or 3 human islet preparations. FACS analysis of mouse islets was performed on three separate occasions, each using a pool of ~300 islets per mouse (B6) collected from 5 or more mice per adenoviral treatment; analysis of human islets was performed on 4 separate human donor preparations, each with >6000 islets per adenoviral treatment. *, *P* < 0.05 relative to LacZ for N ≥ 3. Scale bars in C, 25 μm.

Overexpression of ca-Nfatc2 induced a ~20-fold and ~3-fold increase in the incorporation of [^3^H]-thymidine into DNA in mouse and human islets, respectively (Fig 2A). Whereas ca-Nfatc2 was a very potent mitogen in mouse islets, ca-Nfatc1 did not induce cellular proliferation in mouse islets. In contrast, ca-Nfatc1 was effective to stimulate proliferation in human islets, yielding a ~2-fold increase in [^3^H]-thymidine incorporation into human islet DNA.

To further investigate the effect of the ca-NFAT isoforms on islet cellular proliferation, we analyzed the cell cycle profile of dispersed mouse and human islet cells by flow cytometry. Both ca-Nfatc1 and ca-Nfatc2 significantly increased the proportion of human islet cells in the S-phase of the cell cycle (Fig 2B). In mouse islets, ca-Nfatc2 increased the proportion of cells in S-phase from ~2% to ~14%; ca-Nfatc1 did not stimulate S-phase progression in mouse islets, consistent with our measurements of [^3^H]-thymidine incorporation into DNA in human *vs*. mouse islets (Fig 2A). To identify the cell type within the islet that is induced to replicate by ca-NFAT, we used the incorporation of BrdU into DNA to mark newly proliferated cells, coupled with hormone-specific immunohistochemistry to label α-cells (glucagon) *vs*. β-cells (insulin). As illustrated by the islet sections in Fig 2C, ca-Nfatc1 and ca-Nfatc2 enhanced the proliferation of the β-cells in human islets. In mouse islets, only ca-Nfatc2 was sufficient to promote cellular proliferation, and did so predominantly in β-cells.

To extend our observations that ca-Nfatc2 induces β-cell proliferation, mouse islets were evaluated for two additional markers of proliferation; Ki67, a nuclear antigen present during all phases of the cell cycle, and pHH3 (S10), a selective marker for M-phase progression. Consistent with cell quiescence, control islets showed minimal BrdU incorporation, and no Ki67 (S7A Fig) or pHH3 (S10) (S7B Fig) immunoreactivity. In contrast, ca-NFATc2 induced BrdU incorporation in β-cells, many of which were positive for Ki67 and pHH3 (S10). The expression of 53BP1, a mediator of DNA damage response [18], was not different between control *vs*. Nfatc2-treated islets (S7C Fig). No BrdU^+^/53BP1^+^ nuclei were observed in response to ca-Nfatc2. These results suggest that ca-NFAT expression selectively promotes cellular proliferation through M-phase progression and not DNA-damage repair pathways.

### Constitutively active NFAT stimulates insulin secretion from mouse islets

**Fig 3:**
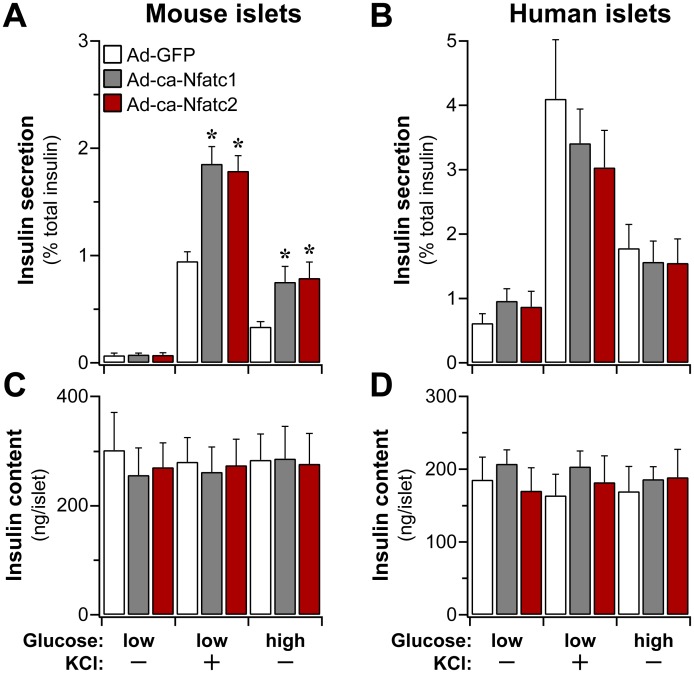
The effect of ca-NFAT on insulin secretion from mouse and human islets. Insulin secretion (**A** and **B**) and islet insulin content (**C** and **D**) were determined from mouse and human islets 72 hr after overexpression of ca-Nfatc1 or ca-Nfatc2 by adenovirus (Ad); Ad-GFP was used as the negative control. Islets were maintained in low glucose (1.7 or 3.3 mM), until changed to media containing secretagogues, as indicated. Insulin secretion was induced with high glucose (16.7 mM) or low glucose plus KCl (40 mM). Secretion is plotted as percent of total insulin present within the islets. All measurements show the mean ± SEM of N ≥ 4 independent mouse or human islet preparations. *, *P* < 0.05 relative to Ad-GFP.

To determine whether ca-NFAT influences β-cell function, we measured insulin secretion following ca-NFAT overexpression. In mouse islets, ca-Nfatc1 and ca-Nfatc2 significantly enhanced insulin secretion in response to high glucose (16.7 mM), or a depolarizing concentration of KCl (40 mM) in the presence of low glucose (Fig 3A). The ca-NFAT-induced increase in insulin secretion from mouse islets was not due to a change in islet insulin content (Fig 3B), suggesting that the insulin secretory cascade was specifically enhanced by ca-NFAT. In contrast to mouse islets, glucose or KCl-induced insulin secretion from human islets was not improved, but more importantly, the overexpression of either ca-NFAT did not cause any functional impairment in islet function (Fig 3C). Total insulin content in human islets was unaffected by ca-NFAT (Fig 3D).

### Constitutively active NFAT regulates gene expression in mouse islets

**Fig 4:**
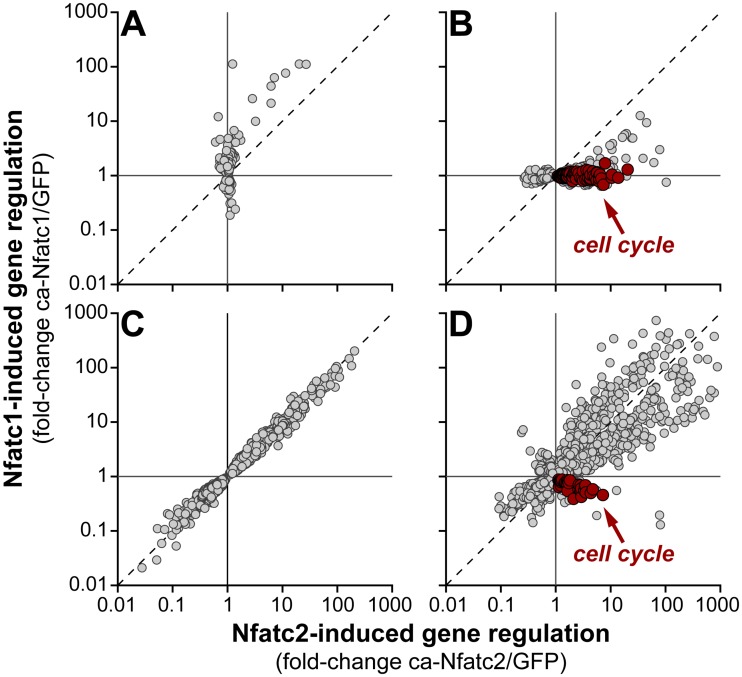
Nfatc1 *vs*. Nfatc2-mediated gene regulation in mouse islets. Whole-islet RNA-sequencing was used to profile gene expression 48 hr after overexpression of ca-Nfatc1, ca-Nfatc2 or GFP (negative control). Genes that were differentially expressed (DE) were classified into one of 4 distinct patterns (relative to GFP): **A**, DE in response to ca-Nfatc1 only (518 genes); **B**, DE in response to ca-Nfatc2 only (1580 genes); **C**, equally DE for ca-Nfatc1 and ca-Nfatc2 (2293 genes); and **D**, unequally DE for ca-Nfatc1 and ca-Nfatc2 (2621 genes). Gene sets enriched with cell cycle regulatory transcripts are highlighted in red. Expression values for all genes and isoforms are contained within S7 Table.

To identify target genes that potentially mediate the effect of NFAT on β-cell proliferation and insulin secretion, we performed RNA sequencing of mouse islets 48 hr after overexpressing ca-Nfatc1, ca-Nfatc2, or GFP. Of the ~20,300 transcripts identified, ~8,800 were differentially expressed (DE) by one or both ca-NFATs compared to GFP. These DE genes tended to follow one of 4 distinct patterns of regulation (Fig 4): **A**) DE in response to ca-Nfatc1 alone; **B**) DE in response to ca-Nfatc2 alone; **C**) DE in response to both ca-Nfatc1 and ca-Nfatc2 equal in magnitude and direction; or **D**) DE in response to both ca-Nfatc1 and ca-Nfatc2, but unequal in magnitude or direction.

Consistent with our observation that only ca-Nfatc2 is a potent β-cell mitogen in mouse islets, one DE gene set (Fig 4B) was highly enriched (*P* < 10^−17^) for genes associated with the cell cycle, including *Mki67*, the cyclins E1, E2 and A2, mini-chromosome maintenance (Mcm) 2, 3, 4, 5 and 7, *Survivin*, *Aurka*, and *Foxm1*. Most of these cell cycle associated genes (~85) included in this DE pattern were induced ~2-fold or more in response to ca-Nfatc2, while remaining unaffected by ca-Nfatc1.

There was one other DE gene set that was significantly enriched for genes associated with the cell cycle (*P* < 10^−4^); genes that were *induced* by ca-Nfatc2, but *suppressed* by ca-Nfatc1 (Fig 4D). This gene set included *Aurkb*, *E2F1*, *3* and *7*, *Tcf19*, *Ezh2*, *Pola1*, and *Cdk4*, among others. The expression of these and ~20 additional cell cycle genes identified in this DE pattern were all suppressed in response to ca-Nfatc1 and induced in response to ca-Nfatc2.

**Fig 5:**
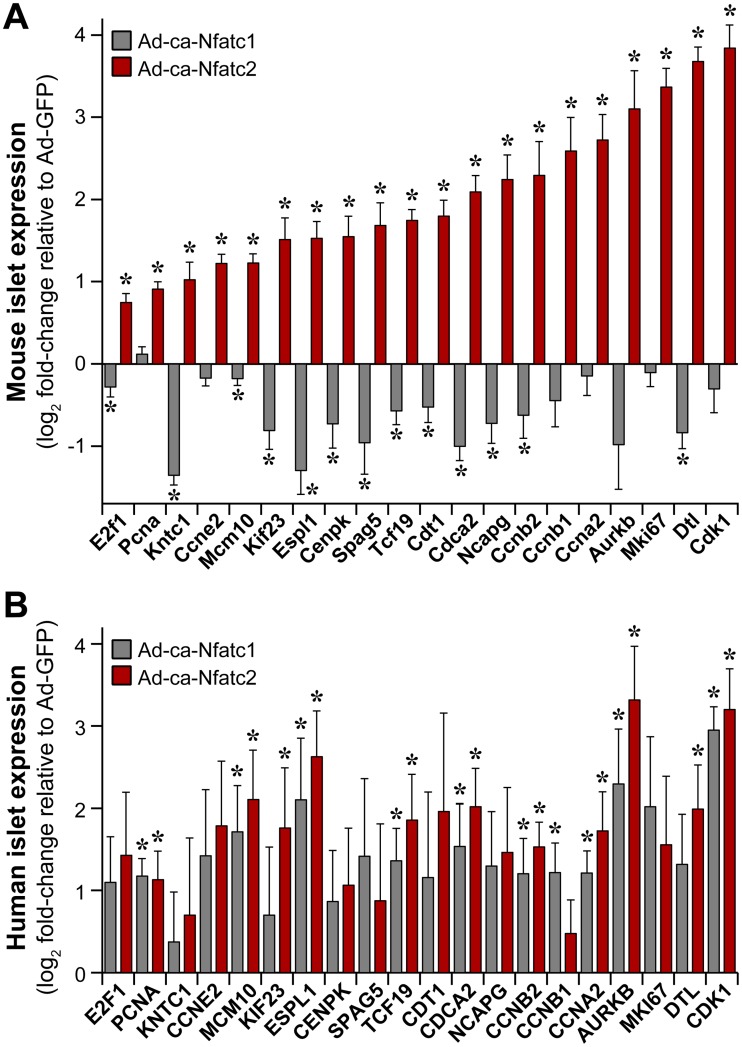
Nfatc1 differentially regulates cell cycle genes in mouse and human islets. The regulation of expression for selected cell cycle genes is illustrated in mouse (**A**) and human (**B**) islets following overexpression of either ca-Nfatc1 or ca-Nfatc2. The data is plotted as the log_2_ fold-change in expression relative to that measured in Ad-GFP (negative control) treated islets. Mouse expression values were obtained from whole-islet RNA-sequencing; human expression values were determined by qPCR. *, *P* < 0.05 relative to negative control. N = 5 for mouse islets; N ≥ 3 for human islets.

To further investigate the significance of the small set of cell cycle genes that were induced by ca-Nfatc2 but suppressed by ca-Nfatc1 in mouse islets (Fig 5A), we asked if they were regulated by the ca-NFAT mutants in human islets (Fig 5B). In human islets, the expression of many of the cell cycle genes was induced by ca-Nfatc1, in parallel with the ability of ca-Nfatc1 to induce β-cell proliferation (Fig 2). In mouse and human islets, these genes were induced by ca-Nfatc2. In addition to the gene expression changes illustrated in Fig 5, several other cell cycle associated transcripts were identified that showed differential regulation by the ca-NFATs in mouse *vs*. human islets, including *Ccnd1*, *Cdkn1c*, *Plk5* and *Nr4a1* (S8 Fig). Some of these genes are known negative regulators of cell cycle progression (e.g., *Plk5*, *Cdkn1c*), and were induced by ca-Nfatc1 in mouse islets, but not in human islets. In summary, one or more of these key cell cycle genes that showed differential regulation by ca-Nfatc1 in mouse and human islets may explain its ability to promote islet cell proliferation.

### Constitutively active NFAT regulates the expression of human T2D GWAS genes in mouse and human islets

**Fig 6:**
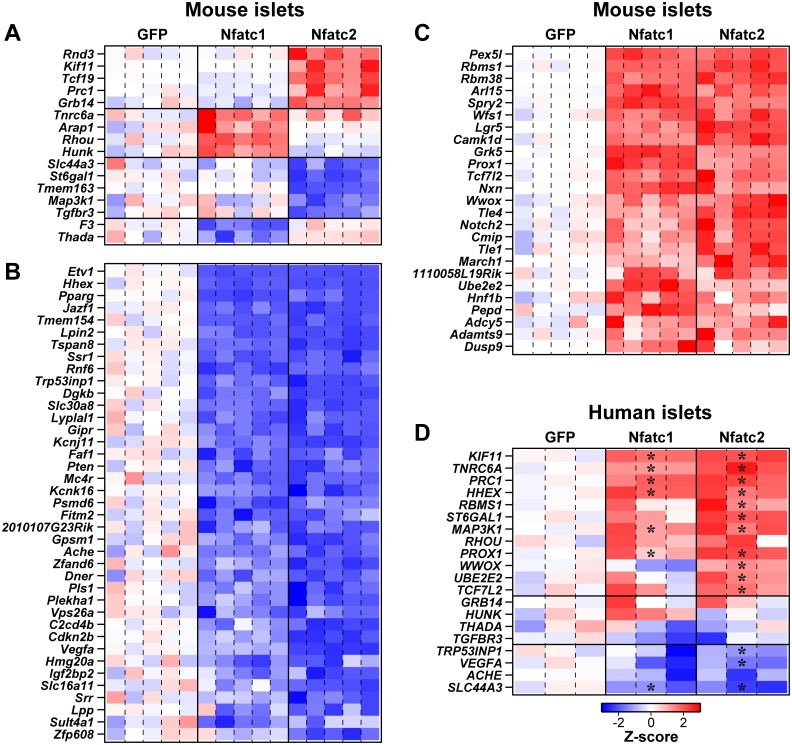
NFAT transcriptionally regulates a T2D GWAS genes in mouse and human islets. Heat maps illustrate the change in the expression of T2D-associated GWAS candidate genes in mouse (**A**, **B** and **C**) and human (**D**) islets in response to the overexpression of ca-Nfatc1, ca-Nfatc2 or GFP. For mouse islets, only those GWAS genes with a posterior probability (*PP*) > 0.99 of being differentially regulated by one or both of ca-NFATs are shown. For human islets, GWAS genes were selected from those showing robust regulation in mouse islets. Gene expression was determined by RNA-sequencing or qPCR in mouse and human islets respectively. Z-scores were computed from expression values for each gene across all samples (15 for mouse and 9 for human), and are shown relative to GFP (average Z-score for GFP = 0), which ranged from -3 to +3. Blue indicates reduced expression; red, increased expression; white, no change. Mouse genes are grouped according to their differential regulation (**A**), versus those that showed roughly equivalent suppression (**B**), or induction (**C**) in response to the two ca-NFATs. In **D**, * indicates *P* < 0.05 for human genes showing differential regulation by ca-Nfatc1 or ca-Nfatc2, relative to GFP.

Ca-NFAT significantly regulated the expression of 80 T2D GWAS gene candidates in mouse islets (Fig 6). Some genes showed exclusive regulation by one isoform (Fig 6A), whereas others are regulated by both isoforms (Fig 6B & 6C). For example, the expression of *Tcf19*, *Kif11*, *Prc1*, *Rnd3*, *Grb14*, *and Cenpw*, all genes associated with cell cycle regulation, were induced by ca-Nfatc2, while remaining unchanged in response to ca-Nfatc1 (Fig 6A). Likewise, *St6gal1*, *Tmem163*, *Tgfbr3*, *Map3k1* and *Tgfbr3* were all exclusively suppressed by ca-Nfatc2. One or more of these genes exclusively regulated by ca-Nfatc2 may be involved in the selective effect we observed for ca-Nfatc2 on β-cell proliferation in mouse islets.

In contrast to genes showing selective regulation by one of the ca-NFATs, many genes were responsive to both NFAT isoforms. For example, the expression of *Tcf7l2*, the gene with the strongest association with human T2D, was induced ~3-fold in response to either of the ca-NFATs (Fig 6C). Other genes induced by both ca-Nfatc1 and ca-Nfatc2, included *Wfs1*, *Camk1d*, and *Lgr5*, whereas *Hhex*, *Slc30a8*, *Tspan8* and *Pparg* were suppressed by both ca-NFATs. One or more of these genes may be involved in the effects that we observed in response to either ca-NFAT on insulin secretion from mouse islets. Of the 50 GWAS genes that showed an islet eQTL on Chr 2, 34 of them (~70%) were transcriptionally regulated by one or both of the ca-NFATs.

We selected a number of GWAS genes that were transcriptionally regulated by one or both of the ca-NFATs in mouse islets, and asked if they were regulated by ca-NFAT in human islets. For our selection, we focused on genes that showed differential regulation by ca-Nfatc1 *vs*. ca-Nfatc2 in mouse islets; i.e., regulated by one isoform, but not the other (Fig 6A), as well as several genes showing equal regulation by the two ca-NFATs (Fig 6B & 6D). Similar to our observations for *TCF19* (Fig 5B), other cell cycle associated genes were upregulated in response to ca-NFAT in human islets. For example, the expression of the cell cycle regulatory genes *KIF11* and *PRC1* was induced by both ca-Nfatc1 and ca-Nfatc2 in human islets (Fig 6D), whereas in mouse islets, these genes were only regulated by ca-Nfatc2 (Fig 6A). Other examples included *SLC44A3* (a member of a choline transporter family) and *PROX1* (a member of the homebox transcription factor family), which were regulated by both ca-NFATs in human islets. Most genes that were suppressed (e.g., *TRP53INP1*, *ACHE*, *VEGA*) or induced (e.g., *UBE2E2*) by ca-NFAT in mouse islets were similarly regulated in human islets (S9 Fig). In summary, our results suggest that a number of the T2D GWAS genes are transcriptional targets (direct or indirect) of the NFAT signaling pathway in both mouse and human islets, and may play a critical role as intermediate traits that mediate the mechanisms by which NFAT affects pancreatic islet function and health.

## Discussion

In T2D, there is insufficient insulin to meet the increased demand resulting from insulin resistance that is usually induced by obesity. The gene candidates near the loci that have been identified in human genetic studies for T2D are thought to predominantly exert their effects on T2D susceptibility in pancreatic islets [1, 5, 19, 20]. Many of these gene candidates control nutrient sensing, insulin secretion, β-cell proliferation, and β-cell survival [21–26].

Our study asked if the expression of the T2D GWAS gene candidates map to specific genomic loci as *trans*-eQTLs in pancreatic islets isolated from an F2 intercross between mouse strains that differ in their susceptibility to diabetes. We show that ~40% of the mRNAs encoded by human T2D GWAS genes mapped as *trans*-eQTLs to a broad region on Chr 2 (Fig 1A). Such co-mapping of the GWAS genes was not observed elsewhere in the genome. All eQTL and diabetes-related clinical data is available at our interactive web site; http://diabetes.wisc.edu/.

The NFAT family of transcription factors is composed of 5 members, Nfatc1-4 and Nfat5, and are expressed in pancreatic islets where they are thought to integrate calcium signals to coordinate gene expression and regulate growth, differentiation and cellular response to environmental cues [27–31]. The NFAT signaling pathway has previously been implicated in the regulation of β-cell development, proliferation and function [32–35]. Cytoplasmic subunits of the Nfatc sub-family are substrates for the calcium-activated serine/threonine phosphatase calcineurin. Calcineurin has been previously shown to be essential for normal β-cell proliferation and function [33–35]. Pancreatic islets express all four Nfatc isoforms. However, their relative abundance and contribution to islet function is not fully understood.

We show that the overexpression of a constitutively active form of *Nfatc2* is sufficient to drive β-cell proliferation in both mouse and human islets (Fig 2), and to stimulate insulin secretion from mouse islets (Fig 3). In addition, *Nfatc2* regulates the islet expression of a large proportion of gene candidates identified in human GWAS as having a genetic association with T2D (Fig 6A–6C). The regulation of these genes by *Nfatc2* may underlie the *trans*-mapping behavior to the *Nfatc2* locus that we observed in our mouse genetic study. In addition to the T2D GWAS genes, our strongest linkage for plasma insulin occurred at this locus. When multiple traits (expression and clinical) co-map to a locus, i.e., demonstrate common genetic architecture, it is possible that one driver mediates the *trans-*linkage of the multiple co-mapping traits. Using *Nfatc2* as a conditional co-variate in our QTL analysis, we show that the LOD profiles for the vast majority of the GWAS genes and plasma insulin were dependent upon *Nfatc2* (Fig 1A & 1B). *Nfatc2* became our top candidate for the driver of the GWAS trans-eQTLs, as its own expression demonstrated a strong *cis*-eQTL, implying local genetic variation directly influenced its expression, along with the GWAS genes and plasma insulin.

We identified a SNP associated with reduced expression of *Nfatc2*, and two coding SNPs that change different amino acid residues in NFAT1, the product of the *Nfatc2* gene, in BTBR mice. Our current study utilized a constitutively active form of NFAT1 that corresponds to the protein sequence for B6 mice. It is possible that these coding differences between the B6 and BTBR NFAT1 proteins influence activation by calcineurin, effective translocation to the nucleus, selective binding to potential nuclear partners for transcriptional gene regulation, or more than one of these steps. Future studies are necessary to delineate the relative contribution of the eQTL SNP *vs*. the coding SNPs on the results reported here.

We show that NFAT regulates the expression of the GWAS genes in human islets (Fig 6D). A number of GWAS genes were similarly regulated by ca-Nfatc1 and ca-Nfatc2 in mouse and human islets (e.g. *RBMS1*, *TP53INP1*, *PROX1*, *TCF7L2*, *HUNK*, *THADA*, *VEGFA* and *SLC44A3*). Those that showed differential regulation in mouse *vs*. human islets, included those associated with the cell cycle; *KIF11*, *PRC1*, *TCF19*. These genes were induced by both ca-NFATs in human, but only by ca-Nfatc2 in mouse, in parallel with enhanced β-cell proliferation. Finally, expression of three GWAS candidates showed opposite regulatory effects in response to ca-Nfatc2 in mouse *vs*. human islets; *ST6GAL1*, *HHEX* and *MAP3K1* were upregulated in human, while down-regulated in mouse in response to ca-Nfatc2. Some of these genes may underlie the differential effects we observed on β-cell proliferation or insulin secretion in mouse *vs*. human islets.

Several groups have recently reported the development of small molecule inhibitors of DYRK1A, the priming kinase that initially phosphorylates the NFATc isoforms (Nfatc1-4), leading to their nuclear exclusion and subsequent inactivation [36–38]. When applied to human or rodent islets, the DYRK1A inhibitors promote β-cell proliferation, and restore normal blood glucose levels in several mouse models of diabetes. These results are consistent with our observations and highlight the importance of the NFAT signaling pathway in regulating β-cell proliferation in rodent and human islets. Interestingly, the DYRK1A inhibitors appear to promote the nuclear accumulation of all four Nfatc isoforms [37, 39]. However, whether one particular isoform mediates the β-cell proliferative effects of the DYRK1A inhibitors, or whether there is redundancy within the NFAT family remains to be determined.

Our study utilized adenovirus to overexpress the ca-NFAT mutants in mouse and human islets. We acknowledge two important points; 1) adenovirus-mediated overexpression typically yields very high levels of expression, raising concerns about non-physiological, off-target effects; and 2) the virus utilized a promoter that does not yield cell-type specific expression, making it difficult to assign NFAT’s action on a particular cell within the islet (e.g., α-cells or β-cells).

We observed isoform-selective effects on β-cell proliferation in mouse islets (Fig 2 and S7 Fig). In mouse islets, both ca-NFATs enhanced glucose and KCl-stimulated insulin secretion, but did not affect basal insulin secretion (Fig 3). Laffitte and colleagues reported that activation of endogenous NFAT signaling in response to small molecule suppression of Dyrk1a in rat islets [37], yielded transcriptional changes similar to what we report here, including cell cycle regulatory genes (*e*.*g*., *Ccna1*, *Aurkb*, *Cdk1*, *Dtl*, *Mki67*, and *Ccnb2*) and T2D GWAS candidates (*e*.*g*., *Pparg*, *Kcnk16*, *Cenpw*, *Tcf19*, *Prc1*, and *Kif11*). Finally, a small collection of cell cycle-related transcripts that were suppressed in mouse islets in response to ca-Nfatc1, were induced in human islets (Fig 5), in parallel with increased cellular proliferation. Our results suggest that despite the high level of expression achieved with adenovirus, the effects we observed on gene regulation and cellular proliferation represent the physiological functions of Nfatc2 in its normal abundance.

Our linkage study, which identified the GWAS hotspot on Chr 2, utilized whole-islet RNA; i.e., islet eQTLs could potentially reflect a specific cell type, or a mix of cells within the islet. It is likely that some of the GWAS eQTLs originate in non-β-cells, for example, *Hhex*, a gene that is selectively expressed in δ-cells [40]. *Hhex* demonstrates linkage to the GWAS hotspot on Chr 2 (S1 Fig), and is transcriptionally regulated by *Nfatc1* and *Nfatc2* in mouse and human islets (Fig 6). Mouse islet expression data recently provided by Huising and colleagues [41] has shown that *Nfatc1* and *Nfatc2* are expressed in all 3 major cell types within the islet; α-cells, δ-cells and β-cells. Thus, using the non-cell type specific promoter to study the ca-NFATs provided a tool to overexpress the construct in all islet cells. Finally, it is also possible that cell-type specific expression of ca-NFAT would nonetheless affect non-transduced cells through the production and secretion of soluble factors, similar to what has recently been reported for β-cell specific expression of *Pdx-1* in rat and human islets [42]. Future studies that would utilize targeted expression of *Nfatc1* and *Nfatc2* are required to fully assess their cell-type specific roles to gene regulation and islet function.

Mouse *Nfatc1* and *Nfatc2* demonstrated an unexpected species selectivity in their ability to promote β-cell proliferation; whereas *Nfatc2* was effective in both mouse and human islets, *Nfatc1* was only effective in human islets (Fig 2). This result runs counter to the widely-held observation that it is easier to induce rodent than human β-cells to proliferate [33, 43–45]. An interesting clue to the underlying mechanism was provided by a small set of cell cycle regulatory genes that were induced by ca-Nfatc2, but suppressed, or not altered, by ca-Nfatc1 in mouse islets (Fig 4D). Some of these genes have been shown to be sufficient to induce β-cell proliferation, including *Ccna2*, *Ccnb1*, *Ccnb2*, *Aurkb* and *Cdk1* [46–48]. That we found these genes to be induced or suppressed in response to ca-Nfatc1 in human *vs*. mouse islets (Fig 5) strongly supports their role in β-cell proliferation. However, future studies are required to determine if the effects we report for *Nfatc2* in mouse islets are sufficient to expand β-cell mass, as has been demonstrated for *Nfatc1* [35].

Using deep RNA-sequencing, we found that multiple isoforms of *Nfatc1* and *Nfatc2* were expressed in mouse islets. The most abundantly expressed *Nfatc1* transcript (variant 1) yields isoform A, a protein consisting of 703 amino acids. In contrast, the most abundantly expressed *Nfatc2* transcripts (variants 1 and 4), yield protein isoforms A and D, consisting of 923 and 927 amino acids respectively. The C-terminal domain of the NFATc protein family is thought to mediate protein-protein interactions with nuclear binding partners, facilitating gene regulation [49–51]. The ca-mutants that we studied correspond to isoform A for both Nfatc1 and Nfatc2. Nfatc2 contains an additional 220 C-terminal amino acids, which may influence which nuclear binding partners are recruited, accounting for the difference in proliferation induction we observed in mouse islets. Kim and colleagues demonstrated that a constitutively active human *NFATC1* isoform containing 716 amino acids successfully restored β-cell mass and cell cycle regulation in a β-cell specific calcineurin knockout mouse [35]. This suggests that species differences may allow for the recruitment of different NFAT nuclear binding partners, and potentially explain why mouse ca-Nfatc2 leads to a ~3 *vs*. ~20-fold increase in β-cell proliferation in human and mouse islets, respectively.

Our data suggests that genes targeted by *Nfatc1* and *Nfatc2* have distinct effects on β-cell proliferation and insulin secretion. Whereas only ca-Nfatc2 induced β-cell proliferation in mouse islets, both ca-NFATs were equally effective in promoting insulin secretion from mouse islets (Fig 3). Several genes known to play a role in the insulin secretory cascade were transcriptionally regulated by the two ca-NFATs, including increased expression of *Tcf7l2*, *Munc13-1*, as well as L-, T- and N-type Ca^2+^ channels, and reduced expression of the K^+^ channel, *Kir6*.*2*, as well as the grehlin receptor, *Ghsr*, and *Ucn3*, both of which have been shown to mediate a negative feedback on insulin secretion between δ-cells and β-cells [41, 52]. These NFAT-induced transcriptional changes may in part underlie some of the stimulatory effects of the ca-NFATs on insulin secretion that we observe in mouse islets.

Neither ca-NFAT isoform exerted a deleterious effect on insulin secretion from human islets (Fig 3C), while significantly enhancing β-cell proliferation (Fig 2). One difference we and others [47, 53] have observed between freshly-isolated rodent islets versus human islets is the level of basal insulin secretion when the islets are maintained in low glucose. In mouse islets, basal insulin secretion is ~0.07 ± 0.02% of insulin content, whereas in human islets basal secretion can be as high as ~0.6 ± 0.1% of insulin content (Fig 3). This nearly 10-fold elevation in basal insulin secretion may in part contribute to a significant difference in islet insulin content observed in human and mouse islets; ~181 and ~275 ng insulin/islet, respectively (*P* < 10^−5^). These differences may have affected our ability to observe a stimulatory effect of the ca-NFATs on insulin secretion from human islets.

Similar to our studies on *Nfatc2*, Newgard and colleagues have reported that the *in vitro* overexpression of the transcription factor *Nkx6*.*1* is sufficient to induce β-cell proliferation and stimulate insulin secretion in isolated rodent and human islets [47]. However, *in vivo* overexpression [54] and inactivation [55] studies have raised questions about Nkx6.1’s role in β-cell proliferation and mass in adult mice. However, recent studies by Sander and colleagues have shown that *Nkx6*.*1* is required for post-natal β-cell expansion [56] and maintenance of β-cell identity [57]. The effects of *Nkx6*.*1* are mediated, at least in part, through two members of the Nr4a nuclear receptor family, *Nr4a1* and *Nr4a3*, which are both necessary and sufficient for *Nkx6*.*1* to regulate β-cell proliferation [47, 58]. In our study of mouse islets, the two ca-NFATs differentially regulated the Nr4a genes; whereas *Nr4a3* was induced by either ca-Nfatc1 or ca-Nfatc2, *Nr4a1* and *Nr4a2* were exclusively induced in response to ca-Nfatc2. Finally, neither ca-NFATc1 nor ca-NFATc2 regulated the expression of *Nkx6*.*1*, suggesting multiple pathways converge to regulate the expression of the Nr4a nuclear receptor gene family.

The GWAS gene candidates were originally identified because their proximity to SNP variants associated with T2D risk. In our mouse islet study, genetic variation occurred at the *Nfatc2* locus, yielding a strong *cis*-eQTL, and not necessarily at the same loci as the human studies. This critical difference suggests that the GWAS gene candidates were responsive to a common regulator, Nfatc2, in our islet eQTL study. Importantly, genetic variation associated with the GWAS genes was not required for our observations. However, the existence of a driver that can regulate a large number of these genes helps explain how the genes might make a substantial contribution to T2D when dysregulated while only making a modest individual contribution when expressed as an allelic variant.

## Methods

High titer, purified adenoviruses containing the well-characterized constitutively active mutants of mouse *Nfatc1* and mouse *Nfatc2* [16, 17], as well as adenoviruses containing GFP or LacZ (used for negative controls) were obtained from Vector Biolabs (Malvern, PA). Genes were overexpressed under the control of the cytomegalovirus (CMV) promoter. [^3^H]-thymidine was purchased from PerkinElmer; Hanks Balanced Salt Solution, penicillin, streptomycin and all tissue culture solutions, from Gibco; RNA isolation kits and PCR reagents from Qiagen; and unless stated otherwise, all other chemicals were from Sigma.

### Construction of B6:BTBR F2 intercross and islet profiling

All animal studies were conducted at the University of Wisconsin, were preapproved by the University’s Research Animal Resource Center, and were in compliance with all NIH animal welfare guidelines. The B6:BTBR F2 intercross was generated as previously described [6]. The *Leptin*^*ob*^ allele was first bred into C57BL/6J (B6) and BTBR *T*^*+*^ *tf*/J (BTBR) mice [59]. B6^*ob/+*^ and BTBR^*ob/+*^ mice were then bred to generate F1^*ob/ob*^ mice. Due to infertility that results from leptin deficiency [60, 61], at 4 weeks of age the F1^*ob/ob*^ mice received subcutaneous transplants of white-adipose tissue from leptin wild-type littermates, allowing us to breed the F1^*ob/ob*^ mice to generate F2^*ob/ob*^ mice. Approximately 550 F2^*ob/ob*^ mice were generated; about half male and half female. All mice were maintained on normal rodent chow diet (Purina 5008) and sacrificed at 10 weeks of age. Pancreatic islets were isolated from the F2^*ob/ob*^ mice as previously described [59] and total RNA was isolated and used for gene expression profiling using a custom Agilent mouse gene expression microarray consisting of ~40,000 60-mer oligonucleotides corresponding to all known genes [6, 59]. Oligonucleotide intensities were normalized to the intensity measured for an islet RNA reference pool that was constructed from all F2^*ob/ob*^ mice, and are reported as the log_10_ of the ratio of each individual mouse relative to the reference pool.

### QTL mapping of islet RNA and plasma insulin

All F2^*ob/ob*^ mice were genotyped with a 5K mouse SNP array (Affymetrix), which identified ~2,000 SNPs that were polymorphic (*i*.*e*., informative) between B6 and BTBR mice, and spread uniformly throughout the mouse genome. These informative SNPs, along with a set of pseudo-markers inserted within intervals flanked by the informative SNPs, were used for QTL mapping of the expression traits as well as plasma insulin as described previously [8]. Expression traits were first transformed into normal quantiles, and then used for single-QTL genome-wide scans [62], allowing for microarray batch and sex as additive and interactive co-variates, respectively. For each expression trait, we focused on the single highest LOD score per chromosome, with a LOD threshold for genome-wide significance of ≥ 5 (*P* < 0.05, trait-wise). Single-QTL analysis was performed on plasma insulin in the same manner as the expression traits, following transformation of the plasma insulin values to normal quantiles.

### Treatment of whole islets with adenovirus for gene overexpression

All human islets were received through the Integrated Islet Distribution Program (IIDP); see S5 Table for donor demographics, as well as the studies conducted on each islet preparation. Upon arrival, human islets were cultured overnight in RPMI containing 8 mM glucose, supplemented with penicillin (100 Units/ml) and streptomycin (100 μg/ml) (Pen/Strep), and 10% heat-inactivated FBS. All mouse islets were harvested from our colony of B6 mice housed within the Biochemistry Department’s vivarium at the University of Wisconsin as described previously [59]. Prior to the application of adenovirus, the islets were treated (~3–4 mins) with a calcium and magnesium-free Hanks Balanced Salt Solution containing 2 mM EGTA. Following exposure to the zero divalent cation solution, adenovirus was applied to the islets in RPMI with 8 mM glucose, supplemented with Pen/Strep, without FBS, for 15 mins in a 200 μl volume, followed by transfer to a 60 mm non-TC treated culture dish containing 3.5 ml of the same culture media (without FBS), and maintained overnight at 37°C. Assuming ~1,000 cells per islet, we added the viruses with an MOI of ~200. Approximately 18 hr after the addition of adenovirus, the islets were washed with fresh media containing RPMI with 8 mM glucose, supplemented with Pen/Strep and 10% heat-inactivated FBS. Islets were maintained in this medium until used as indicated for each study.

### Measures of islet cell proliferation

Four methods were employed to monitor cellular proliferation in mouse and human islets: 1) incorporation of radioactive thymidine into islet DNA; 2) FACS-based separation of disrupted islets into distinct cell cycle phases; 3) incorporation of BrdU into newly replicated islet cells that were co-stained with insulin or glucagon to identify β-cells *vs*. α-cells, respectively; and 4) immuno-cytochemical detection of the proliferation markers, Ki67 or pHH3 (S10). For the thymidine incorporation measure, 1 μCi/ml of [^3^H]-thymidine (PerkinElmer) was added to the culture medium 48 hr after the initial infection of the islets with the adenoviruses. Eighteen hours later, the islets were disrupted and DNA was precipitated with 0.5 ml of ice-cold 10% trichloroacetic acid, followed by re-suspension with 0.1 ml of 0.3 N NaOH. The total amount of [^3^H]-thymidine incorporated into the islet DNA was measured by liquid scintillation counting and normalized to the amount of total cellular protein by Bradford assay.

For the FACS-based separation of the islet cells into respective cell cycle phases, islets were disrupted with the StemPro accutase cell dissociation reagent (Life Technologies, A11105-01) at 37°C for 15 minutes. Islet clusters that remained were gently triturated to facilitate disruption to single cells. Islets cells were transduced with Ad-ca-Nfatc1, Ad-ca-Nfatc2 or Ad-LacZ control viruses at MOI of 2.5 for 4 hr. The disrupted islet cells were re-suspended in culture medium (RPMI medium supplemented with 10% fetal bovine serum and Pen/Strep) and maintained as indicated. Following adenovirus treatment (48 hr), the dispersed human or mouse islet cells were fixed with 100% ethanol and stained with propidium iodide (PI) to provide a quantitative measure of cellular DNA content. Following overnight PI staining (40 μg/ml at 4°C) cells were filtered through a 40-μm sieve, and flow cytometry was performed on a four-laser, LSRII (BD Biosciences). Data were analyzed with ModFit software to estimate the proportion of cells in G0/G1, S and G2/M phases of the cell cycle [63].

To identify the islet cell types that were induced to proliferate in response to ca-NFAT, mouse and human islets were incubated with BrdU (10 μM) added to the culture medium 48 hrs after the initial infection with adenovirus. Islets were removed from culture, washed, and fixed with formalin (10%, 3 hrs, 4°C). The formalin was then removed and the islets were maintained in PBS overnight at 4°C. To aid in their visualization during sectioning, Affi-Gel Blue Gel beads (Biorad) were added to the formalin-fixed islets. The islet and blue bead mixture was fixed in 2% Agar, 1% formalin, followed by paraffin embedding. Sections were dewaxed in xylene (10 mins), rehydrated in decreasing EtOH, boiled in unmasking solution (Vector Labs, H-3300) for 13 mins and then cooled at RT for 10 mins. To reduce non-specific labeling, sections were blocked with Dako Protein Block (X0909) for 30 min, followed by a PBS wash. Guinea pig anti-insulin (Sigma, I-8510), mouse anti-BrdU (Calbiochem, NA-61), goat anti-glucagon (Cell Signaling, 2760S), rabbit anti-Ki67 (Thermo Scientific, 1906s), rabbit anti-pHH3 (S10) (Cell Signaling, 9701S), or rabbit anti-53BP1 (Bethyl Labs, IHC00001) antibodies were added to diluent solution (Dako, S3022), applied to the islet sections and incubated at 4°C overnight. Slides were washed with PBS before the addition of secondary antibodies; Cy3-conjugated anti-guinea pig (Jackson ImmunoResearch); alexafluor 488 anti-rabbit and alexafluor 647 anti-mouse (Life Technologies). The slides were incubated with secondary antibodies (30 min, RT in the dark), washed and then allowed to air dry. Staining was preserved and nuclei identified by adding a drop of Vectashield with DAPI (H-1200, Vector Labs) to each tissue section. Islet sections were imaged with a Nikon AR1 confocal microscope equipped with EZ C1 software (Nikon Corp., Japan).

### Measurement of insulin secretion

To evaluate the effect of ca-NFAT on the function of human and mouse islets, we monitored insulin secretion evoked by a variety of insulin secretagogues, as previously described [64]. Media and cellular insulin was measured by ELISA as described [59]. Cellular insulin is extracted *via* acidified ethanol (0.18 N HCl, 70% EtOH in water). Insulin secretion is expressed as percent of total insulin (media plus cellular insulin values).

### Western blot analysis of NFAT1 and NFAT2 in mouse islets

Islets were collected from ~14 week-old B6 male mice and used for adenoviral transduction using Ad-LacZ, Ad-ca-Nfatc1 or Ad-ca-Nfatc2 as described above. 48 hr after transduction, islets were lysed using RIPA lysis buffer (Abcam, ab156034). Whole islet lysates (40 μg total protein) from each virus treatment were used to determine NFAT2 (*Nfatc1* gene product) and NFAT1 (*Nfatc2* gene product) protein levels; NFAT2 antibody (Thermo, MA3-024), NFAT1 antibody (Cell Signaling, 5861S).

### RNA isolation and qPCR analysis of select gene sets in human islets

Total RNA was isolated from human or mouse islets using the RNeasy spin columns according to the manufacturer’s directions (Qiagen). RNA quantity was determined using a Nanodrop (Thermo Scientific) and the quality assessed by a Bioanalyzer (Agilent). Total mouse RNA was used for RNA-sequencing as described below. Total RNA from human islets was converted to cDNA using a high capacity reverse transcriptase kit (ABI). The cDNA was diluted with water to a final concentration of 4 ng/μl, and used for quantitative gene expression measurement with a Qiagen Rotorgene qRT-PCR machine. Gene-selective primer pairs were generated by “Primer Quest” (IDT). All primer sequences are contained within S6 Table.

### RNA-sequencing and EBSeq analysis in mouse islets

On each of 5 separate days ~1,200 islets were pooled from 6 B6 mice and used for adenoviral treatment as described above to overexpress GFP, ca-Nfatc1 or ca-Nfatc2. 48 hr later, whole islet RNA was isolated using RNeasy purification columns (Qiagen), quantified (Nanodrop) and integrity verified (Agilent) prior to sequencing. The 15 separate RNA samples (N = 5 each for GFP, ca-Nfatc1 and ca-Nfatc2) were bar-coded and randomized for multiplexing across three lanes of an Illumina HiSeq 2000, which yielded ~24M total RNA-sequencing single, paired-end reads/sample (101 bp length). Based on simulation results from Dewey and colleagues, the median percent error is well-controlled (<10%, and in most cases < 5%) with 24M, 101 bp reads in mouse [65]. The RNA-sequencing reads were mapped via bowtie [66] against refseq mm10 reference. Gene and isoform expression values were then estimated via RSEM [65]. Expected counts were normalized using median-by-ratio normalization [67]. Genes and isoform values with 75^th^ quantiles > 10 were used for further analyses.

EB-seq [68], a newly-developed multiple condition model, was used to classify genes and isoforms into 5 distinct patterns of differential expression, DE, (C1, ca-Nfatc1; C2, ca-Nfatc2): Pattern 1: C1 = C2 = GFP (no DE); Pattern 2: C1 ≠ C2 = GFP (DE for C1 only); Pattern 3: C2 ≠ C1 = GFP (DE for C2 only); Pattern 4: C1 = C2 ≠ GFP (DE for C1 and C2 equally); and Pattern 5: C1 ≠ C2 ≠ GFP (DE for C1 and C2 unequally). For each gene or isoform, EB-seq computes a posterior probability (*PP*) associated for each expression pattern. The higher the *PP*(Pattern *k*), the more likely that gene/isoform is following Pattern *k*. To identify DE genes for either C1 or C2, we used a threshold of *PP*(Pattern 1) < 0.01 (*i*.*e*., >99% confidence DE for C1, C2 or both). Genes/isoforms illustrated in Fig 4 have *PP*(Pattern 2–5) > 0.75. S7 Table contains RSEM-normalized expression values, as well as the *PP* values for patterns 1–5 for all genes; raw expression values are also available GEO (GSE73697).

### Quantification of isoform-specific islet gene expression

RNA-Seq data was collected from B6 mouse islets and used to estimate the abundances of endogenously expressed isoforms. Approximately 400 islets were isolated from two B6 mice. Total RNA was isolated from the islets using RNeasy purification columns (Qiagen, Hilden, Germany), verified for integrity on a 2100 Bioanalyzer System (Agilent, Santa Clara, California), and prepared for sequencing using the Illumina TruSeq RNA Sample Prep Rev. A protocol (Illumina, San Diego, CA). Approximately 94 million paired-end reads were sequenced on an Illumina HiSeq 2000 (2 x 101 bp, ~350 bp total size). Isoforms were quantified by running RSEM (version 1.2.4) [65] and Bowtie1 (version 0.12.7) [66] in paired-end mode and using a synthetically reconstructed transcriptome derived from the mm9 reference genome and RefSeq gene models (downloaded from UCSC browser June 24^th^, 2013, NR entries removed). The Bowtie-RSEM pipeline directly maps RNA reads to annotated transcripts (isoforms), which has been show to provide better quantification accuracy for known transcripts compared to pipelines that uses splice aligner to map reads to the whole genome [65, 69]. All other RSEM parameters used were default. Estimated isoform abundances are reported in Transcripts Per Million reads (TPM) [65], as well as Fragments Per Kilobase of transcript per Million mapped reads (FPKM) [70]. The TPM value represents the expected number of reads—per million reads collected—derived from an isoform, as based on the RSEM model of isoform-quantification [65]. Raw expression values are also available at GEO (GSE76477).

### Conditional QTL scans used to support Nfatc2 as the regulator of GWAS genes

To investigate the extent to which variation in *Nfatc2* expression explains variation in GWAS eQTLs, for each GWAS eQTL, we recalculated the LOD score profile while adjusting for *Nfatc2* expression as a covariate. This type of conditional analysis effectively removes the effect of *Nfatc2* on the GWAS eQTLs. To evaluate the specificity of the effect of *Nfatc2* on the GWAS eQTLs, we repeated the conditional analysis for each gene having a significant *cis*-eQTL on Chr 2 (303 *cis*-eQTLs). We determined the number of significant eQTLs (LOD ≥ 5.0) on Chr 2 for all GWAS genes before and after conditioning on each of the *cis*-eQTLs. A summary score was then computed for each *cis*-eQTL based on the reduction in the number of GWAS eQTL following conditioning. S3 Table provides a list of the summary scores for all *cis*-eQTL on Chr 2.

### Accession codes

All expression data has been deposited to the Gene Expression Omnibus (GEO) at NCBI (GSE73697 and GSE76477).

## Supporting Information

S1 Fig T2D GWAS islet eQTLs and plasma insulin QTL that are conditional on Nfatc2. Heat maps show the linkage for plasma insulin and the islet eQTLs for *Nfatc2* and 54 transcripts for genes identified in human GWAS that are associated with Type 2 Diabetes (T2D). Linkage data was obtained from an F2 intercross between diabetes resistant (B6) and diabetes-susceptible (BTBR) mouse strains. Each row shows linkage prior to (**A**) and following (**B**) conditioning on Nfatc2 expression (see methods). Loss of linkage indicates dependence on *Nfatc2*, suggesting *Nfatc2* regulates the expression of the GWAS genes and plasma insulin. Black circles indicate genomic location of genes, and highlight those that demonstrate linkage in *cis* (*e*.*g*., Nfatc2). The majority of the GWAS genes show linkage in *trans*, indicating their expression is regulated by factors present on Chr 2. Color scale by row across (**A**) and (**B**) for LODs ranges from blue (0) to red (max LOD prior to conditioning on *Nfatc2*) for each trait; all traits shown in **A** have max LOD > 5. Red indicates genomic area showing strongest linkage.
(TIF)

S2 Fig Genotype dependence of T2D GWAS *trans*-eQTLs on Chr 2. Expression of GWAS gene candidates in islets of 491 F2 mice. For each gene, mice are grouped according to genotype at the peak locus of the respective eQTL; homozygous B6 (B6:B6), heterozygous (B6:BTBR), or homozygous BTBR (BTBR:BTBR). The expression of 26 GWAS gene candidates increased (**A**) in response to the BTBR allele; 26 GWAS genes decreased with the BTBR allele (**B**). Expression values are the log_10_-transformed ratio for each individual mouse relative to a reference pool constructed from islet mRNA for all mice.
(TIF)

S3 Fig Genotype dependence of expression of *Nfatc2* in pancreatic islets. Expression of *Nfatc2* in pancreatic islets of 491 F2 mice. Mice are grouped according to their genotype at ~168.4 Mb on Chr 2 (rs3024096), the marker position closest to the maximum LOD (~70) of the *cis*-eQTL for *Nfatc2*. At this position, mice were homozygous B6 (B6:B6, N = 127), heterozygous (B6:BTBR, N = 260), or homozygous BTBR (BTBR:BTBR, N = 104). Expression values are the log_10_-transformed ratio for each individual mouse relative to a reference pool constructed from islet mRNA for all mice. Horizontal bars show expression mean ± SEM at each genotype; 0.096 ± 0.008 (B6:B6), -0.017 ± 0.006 (B6:BTBR), and -0.161 ± 0.009 (BTBR:BTBR).
(TIF)

S4 Fig Regional association plots for NFAT and fasting insulin in human GWAS. Association (-log_10_ P-value) to fasting insulin levels for SNPs near *NFATC1* (**A**) and *NFATC2* (**B**). Plots were generated using LocusZoom [13] and data provided in [14]. Color scale shows correlation (r^2^) between the SNP with the strongest association within the plotted region (lead SNP, purple diamond) and other SNPs nearby, defining a haplotype block. For simplicity, SNPs with r^2^ < 0.2 to lead SNP are smaller size. The number of SNPs plotted are 470 and 642 at the *NFATC1* (**A**) and *NFATC2* (**B**) gene loci, respectively. Recombination frequencies are plotted as blue trace and is shown along right-margin.
(TIF)

S5 Fig Sequence comparison of mouse and human Nfatc1 and Nfatc2. Amino acid sequence for mouse and human, proteins for equivalent isoforms of Nfatc2 (**A**) and Nfatc1 (**B**) were aligned using Clustal Omega. For Nfatc2, we used isoforms A (NP_035029.2) and C (NP_775114.1) for mouse and human, respectively. For Nfatc1, we used isoforms 1 (NP_058071.2) and I (NP_001265604.1) for mouse and human, respectively [71]. The calcineurin binding site (cyan), Ser residues changed to Ala residues in the ca-mutants (red), and Rel homology domain (yellow) are shown. Identical (*), conserved (:), and similar (.) residues are indicated.
(TIF)

S6 Fig Expression of the NFAT gene family in mouse islets transduced with adenoviruses. Normalized RNA-sequencing values for the NFAT gene family in mouse islets 48 hr after transduction with adenoviruses containing GFP, ca-Nfatc1 or ca-Nfatc2 (**A**). Average expression values (± S.E.M., N = 5) are shown for each gene/virus combination. Western blot analysis for native NFAT2 (left panel) and NFAT1 (right panel) proteins, gene products for *Nfatc1* and *Nfatc2*, respectively, in mouse islets 48 hr after transduction with indicated adenoviruses (**B**). MW standards (tick marks) were 75 and 100 kDa (NFAT2 blot), and 100 and 150 kDa (NFAT1 blot).
(TIF)

S7 Fig The overexpression of ca-Nfatc2 promotes cell cycle progression and not DNA damage repair pathways in mouse islets. Immunocytochemistry of mouse islets for (**A**) Ki67, (**B**) pHH3 (S10) and (**C**) 53BP1 following Ad-LacZ (control) and Ad-ca-Nfatc2 transduction (72 hr). To identify β-cells, islets were stained for insulin. All islets were exposed to BrdU (18 hr) to monitor proliferation. White-green arrowheads indicate BrdU/Ki67 or BrdU/pHH3 (S10) dual-positive nuclei. Monochrome arrowheads identify nuclei expressing only one marker. Scale bar = 10 μm. Immunofluorescent images are representative of >60 islets photographed from 4 different mouse islet preparations per adenoviral treatment.
(TIF)

S8 Fig Additional cell cycle regulatory genes that were differentially regulated by ca-Nfatc1 in mouse and human islets. The regulation of expression for cell cycle genes is illustrated in mouse (**A**) and human (**B**) islets following overexpression of either ca-Nfatc1 or ca-Nfatc2. The data is plotted as the log_2_ fold-change in expression relative to that measured in Ad-GFP (negative control) treated islets. Mouse expression values were obtained from whole-islet RNA-sequencing; human expression values were determined by qPCR. *, *P* < 0.05 relative to negative control. N = 5 for mouse islets; N = 8 for human islets.
(TIF)

S9 Fig Comparison of GWAS genes that were regulated by NFAT in mouse and human islets. Heat maps illustrate the change in the expression of T2D-associated GWAS gene candidates in mouse (left) and human (right) islets replotted from Fig 6 as the average Z-score for each transcript; N = 5 for mouse and N = 3 for human.
(TIF)

S1 Table Gene candidates linked to T2D risk loci from human GWAS, and their mouse homologues. Separate tabs list: Tab 1, ~300 entries in the GWAS catalog for genomic loci associated with the disease/trait “Type 2 diabetes”; Tab 2, distinct genomic loci and their associated P-values; and Tab 3, candidate human genes reported for the loci, with accompanying mouse homologues obtained from http://www.informatics.jax.org.
(XLSX)

S2 Table Islet eQTLs for T2D GWAS candidates in mouse islets. Excel sheet lists the 205 eQTLs for GWAS candidates genes that were identified genome-wide in islets from ~500 B6:BTBR-F2 obese mice. Genomic positions for the genes and their eQTLs are shown. *Cis* is defined as an eQTL that occurred within 2.5 cM (~5 Mbp) of the genomic position of the corresponding gene; all others are classified as *trans*-eQTLs. LOD scores for eQTLs are shown before and after conditioning on the *cis*-eQTL for Nftac2 and are colored by the magnitude of change; blue decrease, red increase, white no change.
(XLSX)

S3 Table Summary scores for islet transcription factor *cis*-eQTLs on chromosome 2 for conditional dependence on T2D GWAS gene candidates. Table summarizing conditional dependence of T2D GWAS gene candidates on islet Chr 2 *cis*-eQTLs for genes annotated as playing a role in "transcription" or "DNA binding" (https://david.ncifcrf.gov/). For each *cis*-eQTL, the score indicates the number of T2D GWAS gene eQTLs before *vs*. after conditioning. Genes with the lowest score (most negative) showed the greatest reduction in GWAS eQTLs. Nfatc2 was the top-ranked transcription factor on Chr 2.
(XLSX)

S4 Table Isoform-specific expression of the NFAT gene family in mouse islets. Islets from B6 mice were used for deep RNA-sequencing to determine isoform-specific expression of all genes. The table shows the expression level and relative proportion of each isoform for the NFAT gene family. Expression values for all genes is available at GEO submission GSE73697.
(XLSX)

S5 Table Donor information for human islet preparations. For several islet preparations, multiple studies were conducted, which are listed in the final column labeled “Experiments”. Values that are missing are not known.
(PDF)

S6 Table Quantitative real time PCR primers used for gene expression measurements in human islets. (XLSX)

S7 Table Normalized expression values for all genes from RNA-sequencing of mouse islets following overexpression of GFP, ca-Nfatc1 or ca-Nfatc2. Excel spreadsheet contains normalized expression values for all genes (Tab 1) identified in mouse islets 48 hr after overexpression of GFP, ca-Nfatc1 or ca-Nfatc2 (N = 5 ea.). Posterior probabilities (*PP*) for differential expression (DE) were computed for each identified transcript to following one of 5 patterns of expression (C1, ca-Nfatc1; C2, ca-Nfatc2): Pattern 1: C1 = C2 = GFP (no DE); Pattern 2: C1 ≠ C2 = GFP (DE for C1 only); Pattern 3: C2 ≠ C1 = GFP (DE for C2 only); Pattern 4: C1 = C2 ≠ GFP (DE for C1 and C2 equally); and Pattern 5: C1 ≠ C2 ≠ GFP (DE for C1 and C2 unequally). To improve model fitting for *PP* determination, transcripts with 75^th^ quantile expression values < 10 have *PP* set to “NA”. Tab 2 contains those genes determined to be DE, defined as *PP*(1) < 0.01.
(XLSX)
