# Supplementary figures and images for: The Transcription Factor Nfatc2 Regulates β-Cell Proliferation and Genes Associated with Type 2 Diabetes in Mouse and Human Islets
Source: PLoS Genet. 2016 Dec 9;12(12):e1006466. doi: 10.1371/journal.pgen.1006466 (PMC5147809; doi:10.1371/journal.pgen.1006466)

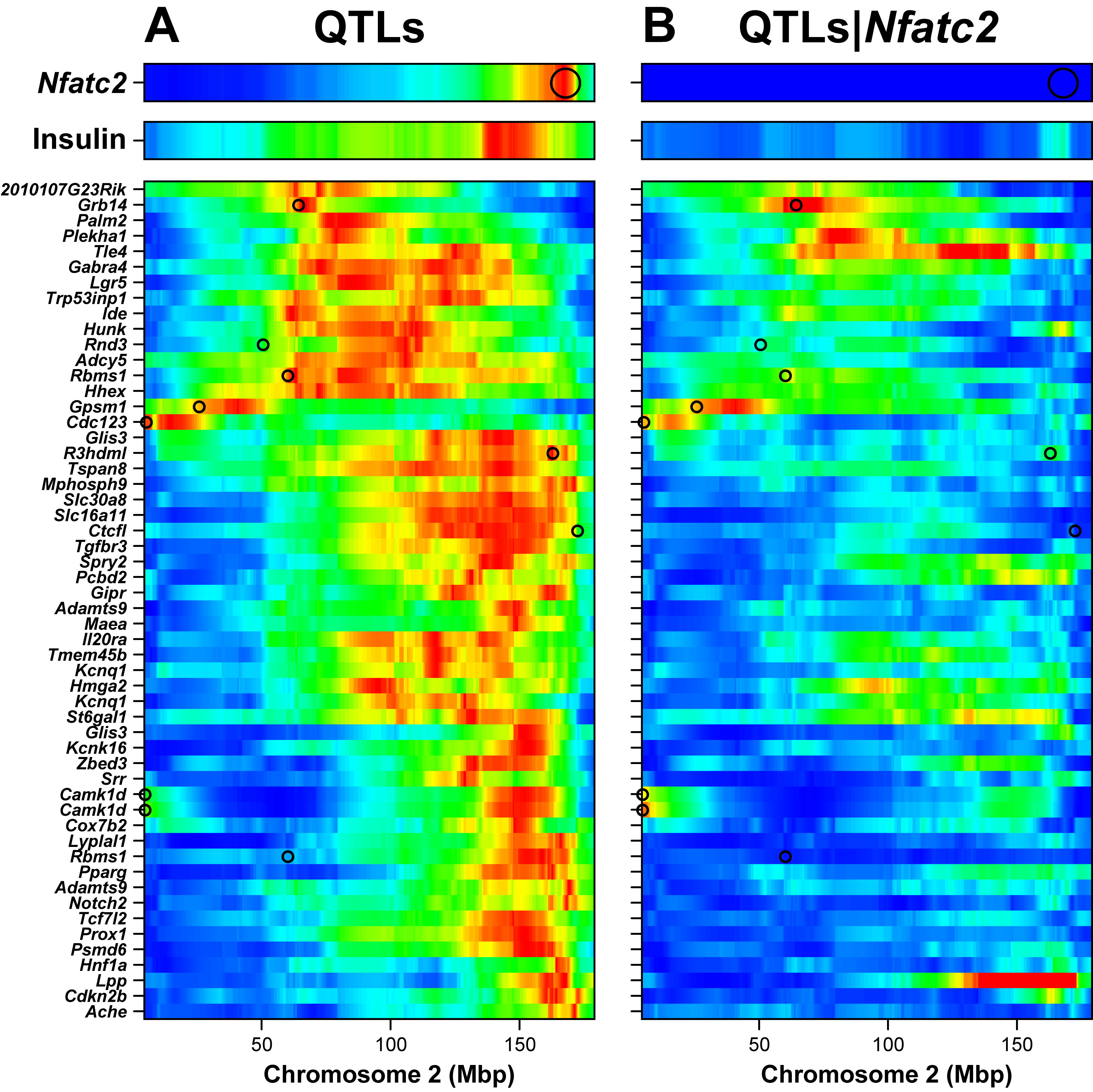

Supplement: S1 Fig — Heat maps show the linkage for plasma insulin and the islet eQTLs for Nfatc2 and 54 transcripts for genes identified in human GWAS that are associated with Type 2 Diabetes (T2D). Linkage data was obtained from an F2 intercross between diabetes resistant (B6) and diabetes-susceptible (BTBR) mouse strains. Each row shows linkage prior to (A) and following (B) conditioning on Nfatc2 expression (see methods). Loss of linkage indicates dependence on Nfatc2, suggesting Nfatc2 regulates the expression of the GWAS genes and plasma insulin. Black circles indicate genomic location of genes, and highlight those that demonstrate linkage in cis (e.g., Nfatc2). The majority of the GWAS genes show linkage in trans, indicating their expression is regulated by factors present on Chr 2. Color scale by row across (A) and (B) for LODs ranges from blue (0) to red (max LOD prior to conditioning on Nfatc2) for each trait; all traits shown in A have max LOD > 5. Red indicates genomic area showing strongest linkage. (TIF) [file pgen.1006466.s001.tif]

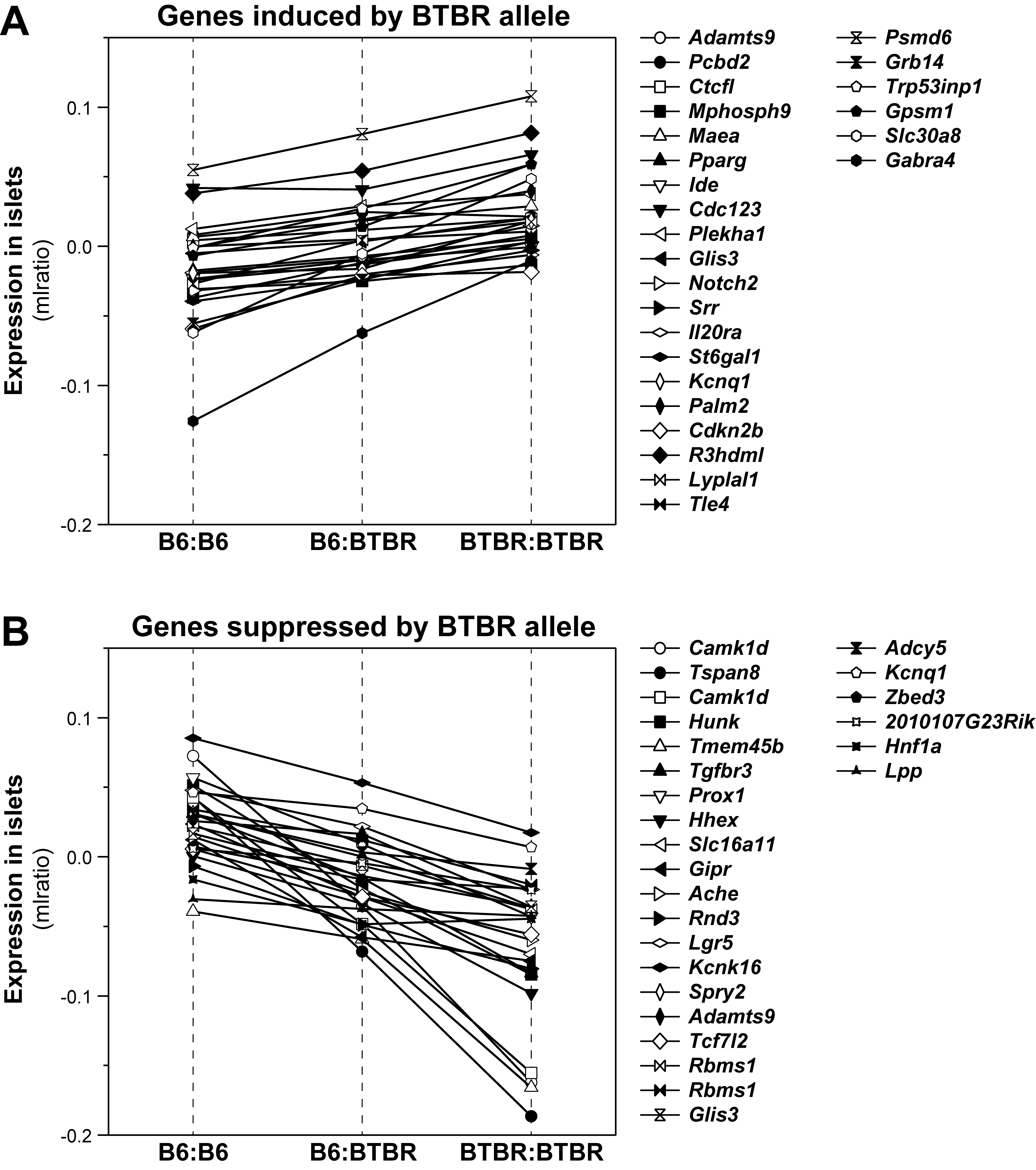

Supplement: S2 Fig — Expression of GWAS gene candidates in islets of 491 F2 mice. For each gene, mice are grouped according to genotype at the peak locus of the respective eQTL; homozygous B6 (B6:B6), heterozygous (B6:BTBR), or homozygous BTBR (BTBR:BTBR). The expression of 26 GWAS gene candidates increased (A) in response to the BTBR allele; 26 GWAS genes decreased with the BTBR allele (B). Expression values are the log10-transformed ratio for each individual mouse relative to a reference pool constructed from islet mRNA for all mice. (TIF) [file pgen.1006466.s002.tif]

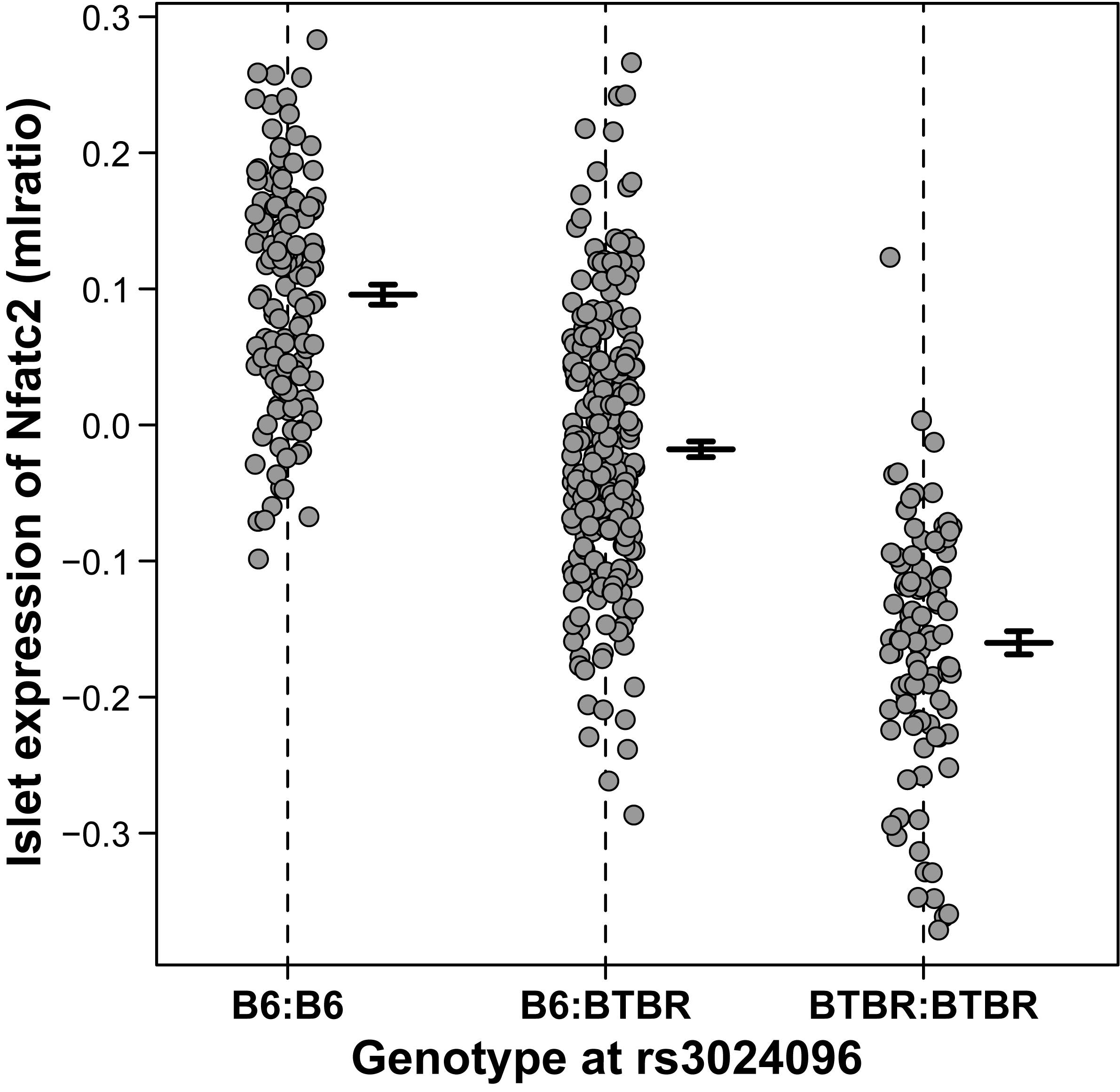

Supplement: S3 Fig — Expression of Nfatc2 in pancreatic islets of 491 F2 mice. Mice are grouped according to their genotype at ~168.4 Mb on Chr 2 (rs3024096), the marker position closest to the maximum LOD (~70) of the cis-eQTL for Nfatc2. At this position, mice were homozygous B6 (B6:B6, N = 127), heterozygous (B6:BTBR, N = 260), or homozygous BTBR (BTBR:BTBR, N = 104). Expression values are the log10-transformed ratio for each individual mouse relative to a reference pool constructed from islet mRNA for all mice. Horizontal bars show expression mean ± SEM at each genotype; 0.096 ± 0.008 (B6:B6), -0.017 ± 0.006 (B6:BTBR), and -0.161 ± 0.009 (BTBR:BTBR). (TIF) [file pgen.1006466.s003.tif]

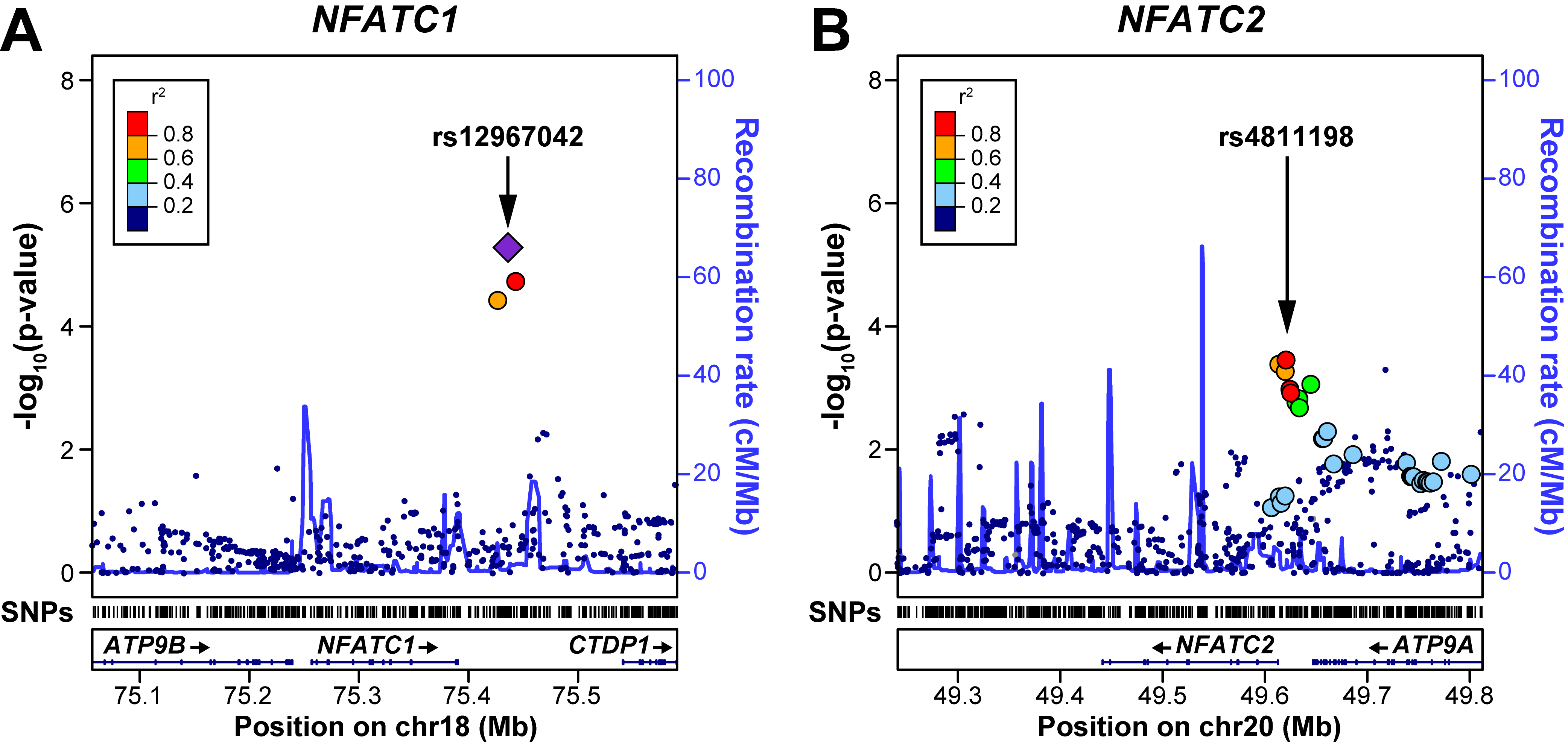

Supplement: S4 Fig — Association (-log10 P-value) to fasting insulin levels for SNPs near NFATC1 (A) and NFATC2 (B). Plots were generated using LocusZoom [13] and data provided in [14]. Color scale shows correlation (r2) between the SNP with the strongest association within the plotted region (lead SNP, purple diamond) and other SNPs nearby, defining a haplotype block. For simplicity, SNPs with r2 < 0.2 to lead SNP are smaller size. The number of SNPs plotted are 470 and 642 at the NFATC1 (A) and NFATC2 (B) gene loci, respectively. Recombination frequencies are plotted as blue trace and is shown along right-margin. (TIF) [file pgen.1006466.s004.tif]

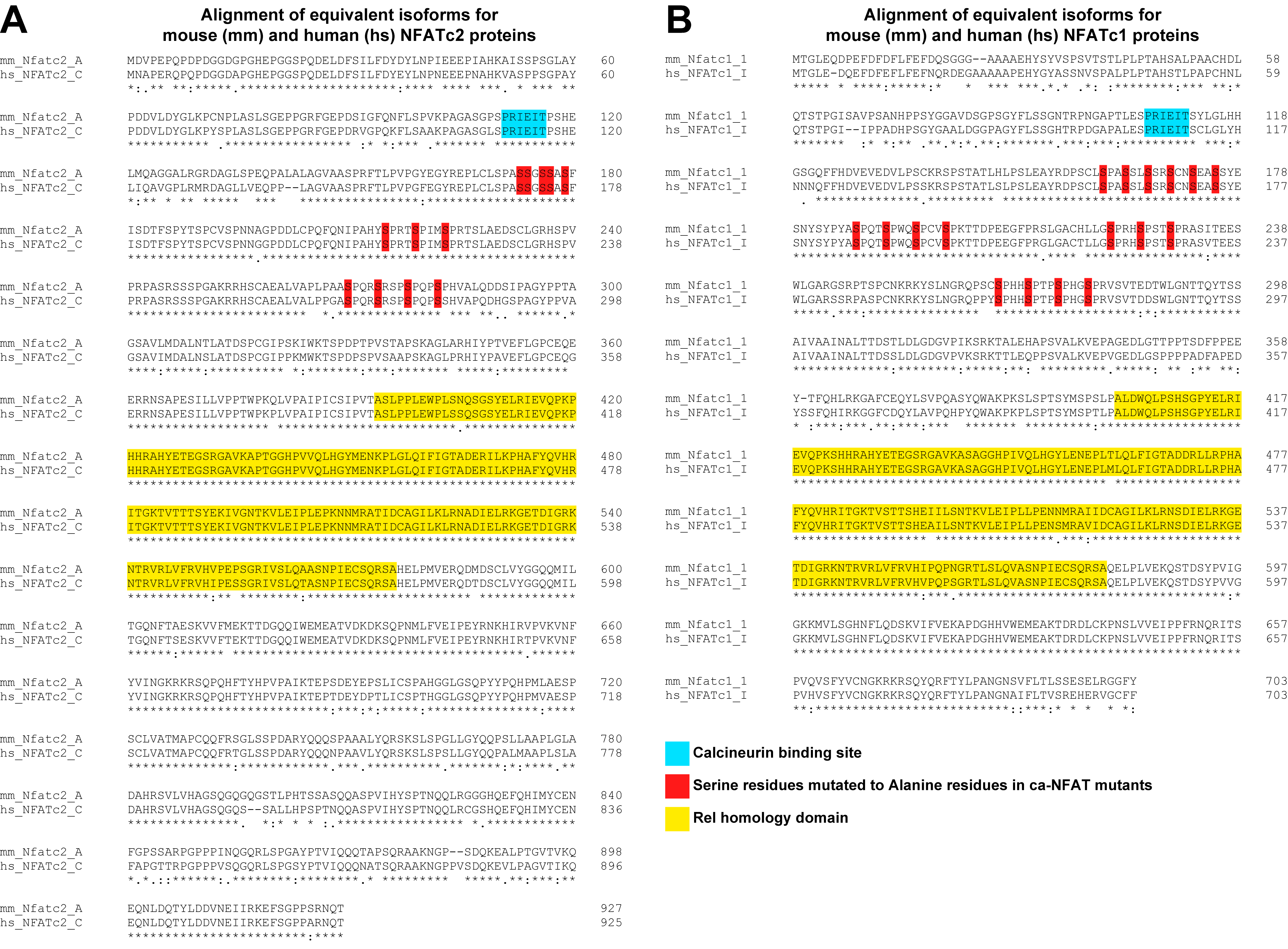

Supplement: S5 Fig — Amino acid sequence for mouse and human, proteins for equivalent isoforms of Nfatc2 (A) and Nfatc1 (B) were aligned using Clustal Omega. For Nfatc2, we used isoforms A (NP_035029.2) and C (NP_775114.1) for mouse and human, respectively. For Nfatc1, we used isoforms 1 (NP_058071.2) and I (NP_001265604.1) for mouse and human, respectively [71]. The calcineurin binding site (cyan), Ser residues changed to Ala residues in the ca-mutants (red), and Rel homology domain (yellow) are shown. Identical (*), conserved (:), and similar (.) residues are indicated. (TIF) [file pgen.1006466.s005.tif]

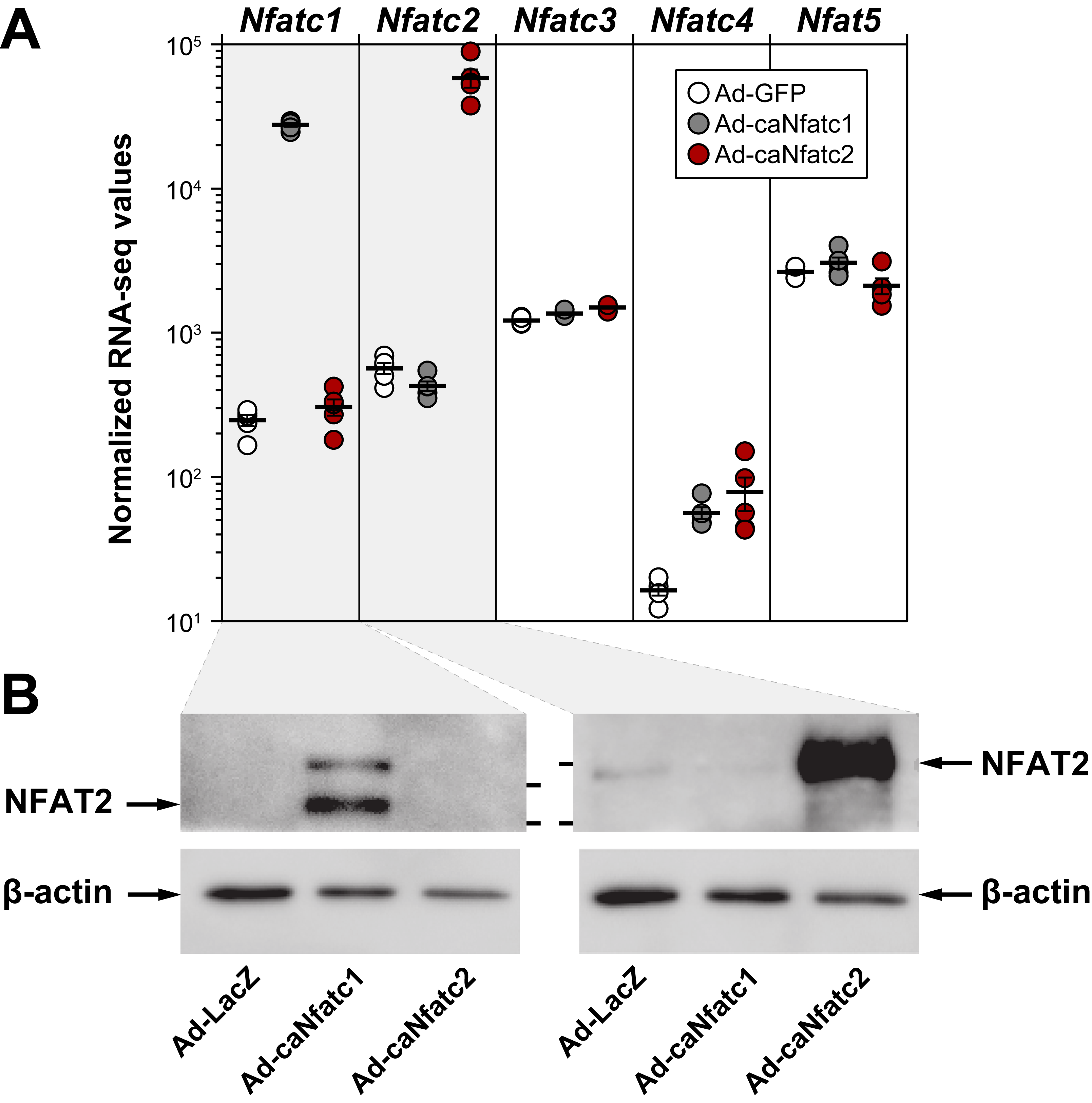

Supplement: S6 Fig — Normalized RNA-sequencing values for the NFAT gene family in mouse islets 48 hr after transduction with adenoviruses containing GFP, ca-Nfatc1 or ca-Nfatc2 (A). Average expression values (± S.E.M., N = 5) are shown for each gene/virus combination. Western blot analysis for native NFAT2 (left panel) and NFAT1 (right panel) proteins, gene products for Nfatc1 and Nfatc2, respectively, in mouse islets 48 hr after transduction with indicated adenoviruses (B). MW standards (tick marks) were 75 and 100 kDa (NFAT2 blot), and 100 and 150 kDa (NFAT1 blot). (TIF) [file pgen.1006466.s006.tif]

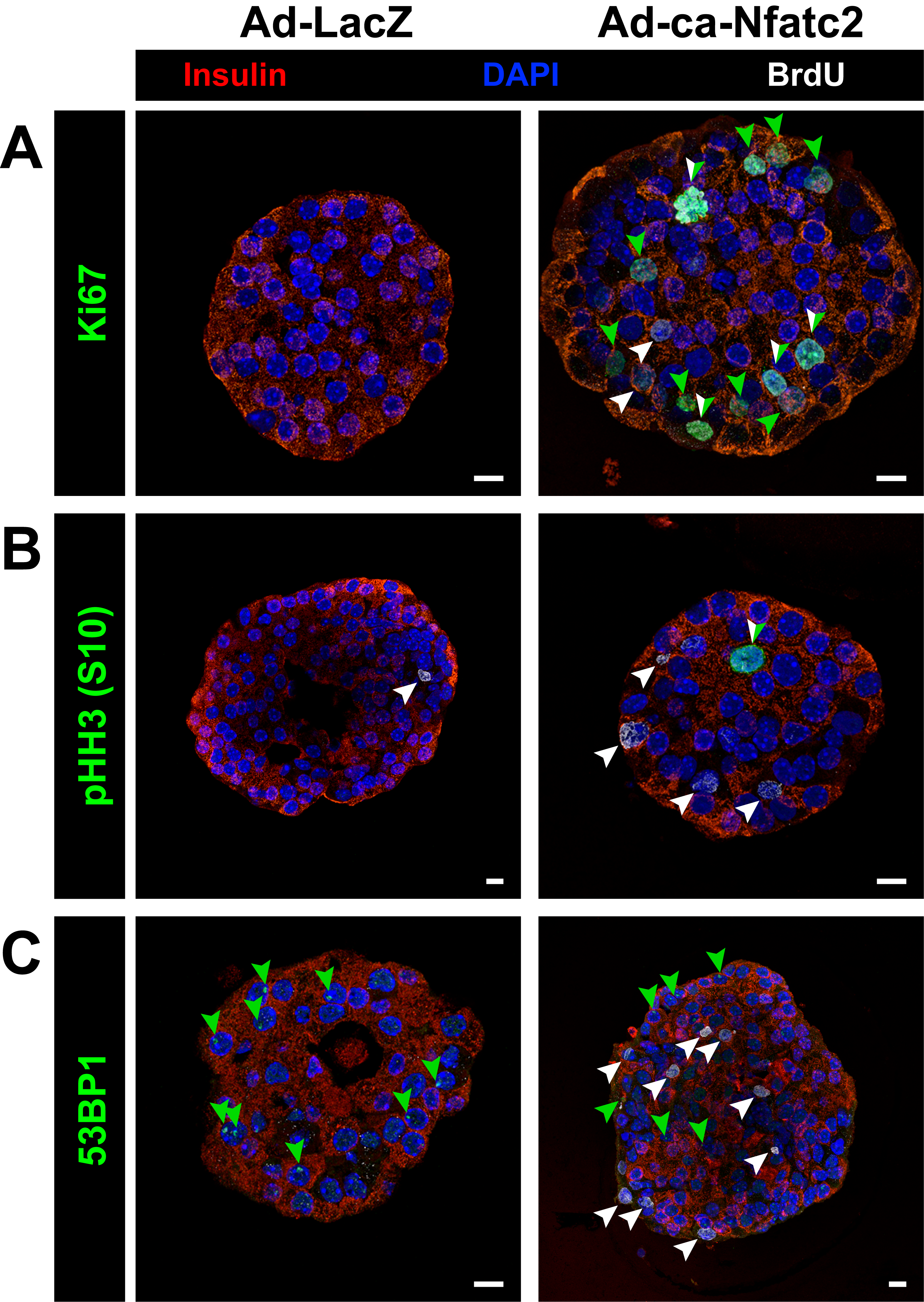

Supplement: S7 Fig — Immunocytochemistry of mouse islets for (A) Ki67, (B) pHH3 (S10) and (C) 53BP1 following Ad-LacZ (control) and Ad-ca-Nfatc2 transduction (72 hr). To identify β-cells, islets were stained for insulin. All islets were exposed to BrdU (18 hr) to monitor proliferation. White-green arrowheads indicate BrdU/Ki67 or BrdU/pHH3 (S10) dual-positive nuclei. Monochrome arrowheads identify nuclei expressing only one marker. Scale bar = 10 μm. Immunofluorescent images are representative of >60 islets photographed from 4 different mouse islet preparations per adenoviral treatment. (TIF) [file pgen.1006466.s007.tif]

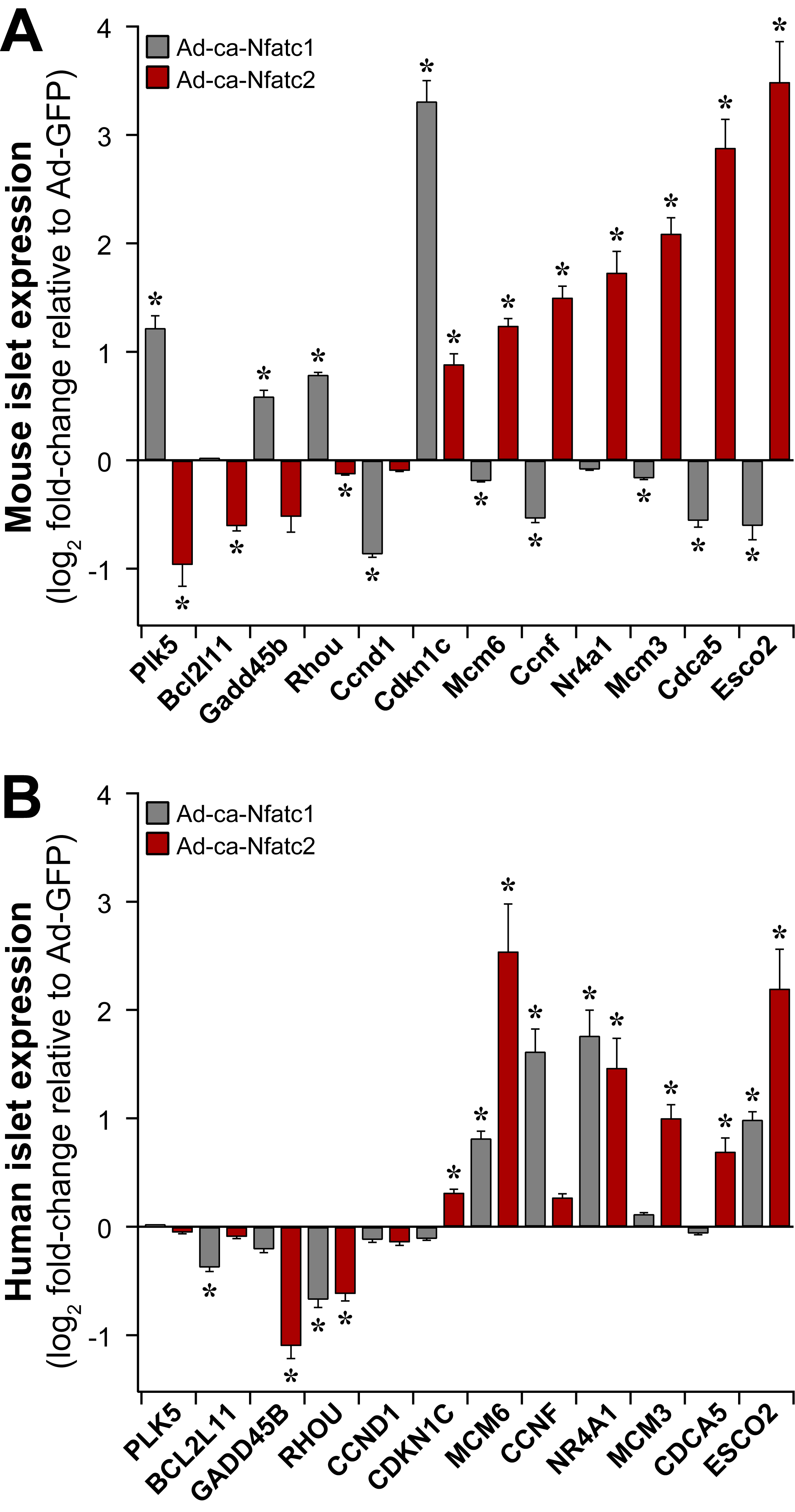

Supplement: S8 Fig — The regulation of expression for cell cycle genes is illustrated in mouse (A) and human (B) islets following overexpression of either ca-Nfatc1 or ca-Nfatc2. The data is plotted as the log2 fold-change in expression relative to that measured in Ad-GFP (negative control) treated islets. Mouse expression values were obtained from whole-islet RNA-sequencing; human expression values were determined by qPCR. *, P < 0.05 relative to negative control. N = 5 for mouse islets; N = 8 for human islets. (TIF) [file pgen.1006466.s008.tif]

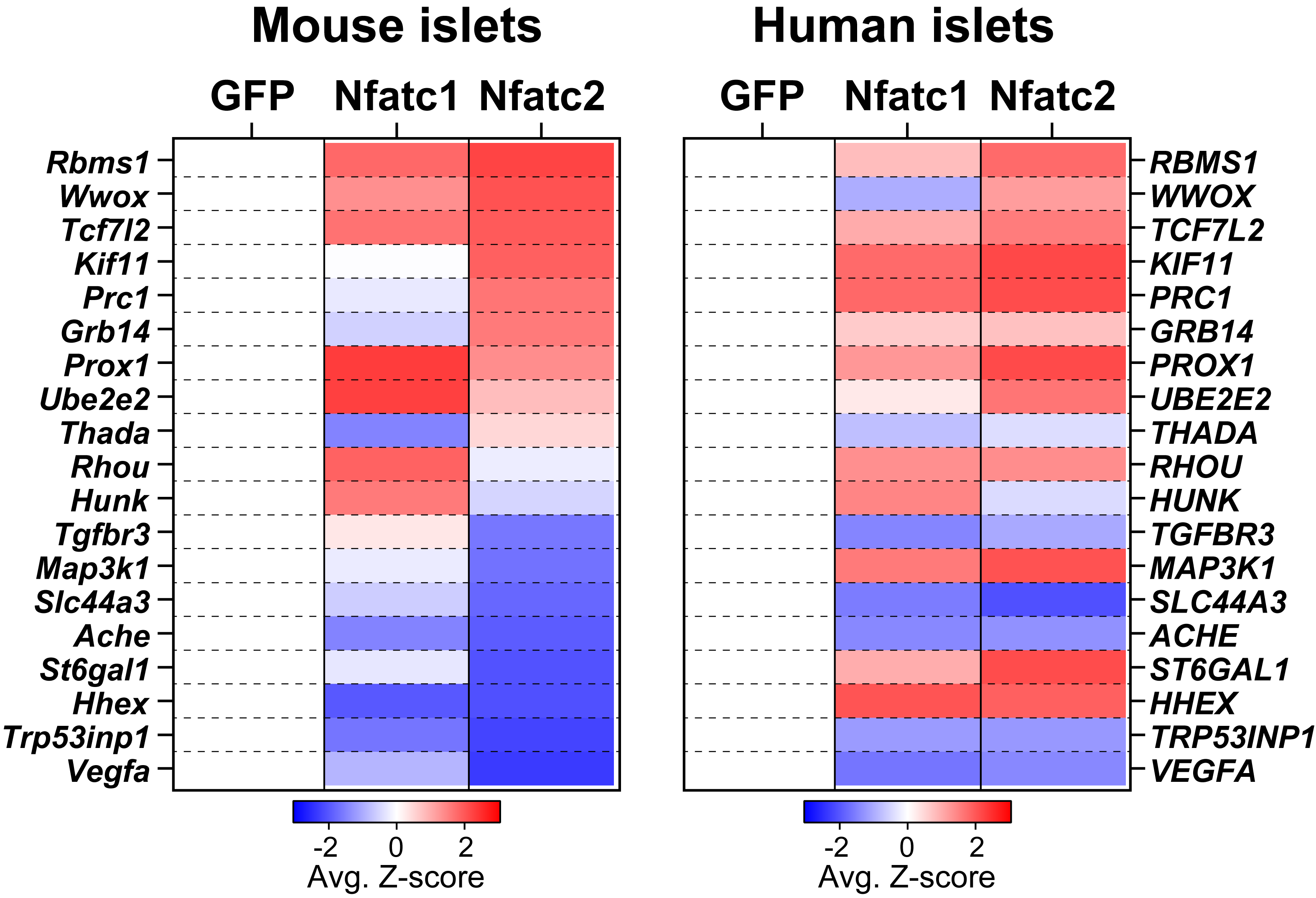

Supplement: S9 Fig — Heat maps illustrate the change in the expression of T2D-associated GWAS gene candidates in mouse (left) and human (right) islets replotted from Fig 6 as the average Z-score for each transcript; N = 5 for mouse and N = 3 for human. (TIF) [file pgen.1006466.s009.tif]
