# Supplementary material for: The Transcription Factor Nfatc2 Regulates β-Cell Proliferation and Genes Associated with Type 2 Diabetes in Mouse and Human Islets
Source: PLoS Genet. 2016 Dec 9;12(12):e1006466. doi: 10.1371/journal.pgen.1006466 (PMC5147809; doi:10.1371/journal.pgen.1006466)
Supplement: S5 Table — For several islet preparations, multiple studies were conducted, which are listed in the final column labeled “Experiments”. Values that are missing are not known. (PDF) [file pgen.1006466.s014.pdf]

Date	Institution	BMI	Age	Sex	Race	CI (min)	CT (hrs)	Purity	Viability	Type	Assays
**2/22/2013**	The Sharp/Lacy Institute	25.7	59	F	Black or African American	911	48	95%	95%	ND	GSIS
**3/27/2013**	Miami	20.2	27	M	White	650	45	85%	94%	ND	GSIS
**5/7/2014**	Wisconsin	36.4	45	M	White	170	24	90%	95%	ND	3H-Thy, FACS
**5/16/2014**	Miami	28.7	32	F	White	843	18	80%	94%	ND	3H-Thy, FACS, IHC
**6/17/2014**	The Sharp/Lacy Institute	31	48	F	American Indian/Alaska Native	372	72	90%	98%	ND	GSIS, 3H-Thy, FACS
**7/14/2014**	SC-ICRC	33.8	25	M	Hispanic/Latino		72	85%	95%	ND	GSIS
**7/14/2014**	Miami	41.3	50	F	Hispanic/Latino	319	24	87%	95%	ND	GSIS
**8/18/2014**	The Sharp/Lacy Institute	34.9	63	F	White	497	88	85%	95%	ND	GSIS
**9/8/2014**	Pennsylvania	34.3	45	F	White	735	71	80%	95%	ND	GSIS, FACS, IHC
**11/10/2014**	The Sharp/Lacy Institute	36.2	34	F	Hispanic/Latino	749	98	90%	95%	ND	IHC
**12/10/2014**	The Sharp/Lacy Institute	32.4	45	M	White	540	51	90%	95%	ND	IHC
**12/16/2014**	Pennsylvania	36.5	40	M	White	425	39	80%	93%	ND	IHC
**2/22/2013**	The Sharp/Lacy Institute	25.7	59	F	Black or African American	911	48	95%	95%	ND	GSIS
**1/7/2016**	University of Illinois-Chicago	26.6	45	F	White	527	51	90%	97%	ND	RT-qPCR
**2/9/2016**	The Sharp/Lacy Institute	24.4	43	M	Pakistani	674	97	95%	95%	ND	RT-qPCR
**2/18/2016**	Wisconsin	56.8	30	M	White	480	19	95%	98%	ND	RT-qPCR
**2/23/2016**	The Sharp/Lacy Institute	21.9	63	M	White	593	68	95%	95%	ND	RT-qPCR
**3/1/2016**	Pennsylvania	40.2	24	F	White	737	92	90%	92%	ND	RT-qPCR
**4/12/2016**	Wisconsin	30.1	54	F	White	675	22	95%	98%	ND	RT-qPCR
**4/26/2016**	The Sharp/Lacy Institute	29.6	39	F	Hispanic/Latino	668	108	95%	95%	ND	IHC
**5/31/2016**	Wisconsin	34.2	33	F	Black or African American	855	42	95%	98%	ND	RT-qPCR
**6/7/2016**	SC-ICRC	23.0	40	F	Hispanic/Latino		51	90%	96%	ND	RT-qPCR

Abbreviations. CI, cold ischemia time; CT, time in culture prior to shipment to UW-Madison; ND, non-diabetic donor; Assays: GSIS, glucose-stimulated insulin secretion studies; 3H-Thy, [3H]-thymidine measurement of proliferation; FACS, fluorescence activated cell sorting measure of proliferation; IHC, immunohistochemistry; qPCR, quantitative gene expression measurement. If a value is missing, it was not known.
